# Effects of Probiotics and Vitamin D Deficiency Correction on Clinical, Inflammatory, Metabolic, and Microbiota Profiles in Older Adults with Oral Lichen Planus: A Multi-Omics Study

**DOI:** 10.3390/ijms27156707

**Published:** 2026-07-27

**Authors:** Paola Zanetta, Matteo Calgaro, Marta Mellai, Alessia Vignoli, Monica Marotta, Nicola Vitulo, Leonardo Tenori, Marcello Manfredi, Elettra Barberis, Mario Migliario, Marta Armari, Valeria Caneparo, Diletta Francesca Squarzanti, Marta Allesina, Angela Amoruso, Marco Pane, Barbara Azzimonti

**Affiliations:** 1 Laboratory of Applied Microbiology, Center for Translational Research on Allergic and Autoimmune Diseases (CAAD), Department of Health Sciences (DiSS), School of Medicine, Università del Piemonte Orientale (UPO), Corso Trieste 15/A, 28100 Novara, Italy; paola.zanetta@uniupo.it (P.Z.); marta.armari@uniupo.it (M.A.); diletta.squarzanti@med.uniupo.it (D.F.S.); 2 Department of Biotechnology, University of Verona, Strada le Grazie, 15, 37134 Verona, Italy; matteo.calgaro_01@univr.it (M.C.); nicola.vitulo@univr.it (N.V.); 3 Integrative Genomics Core Facility, Center for Translational Research on Allergic and Autoimmune Diseases (CAAD), Department of Health Sciences (DiSS), School of Medicine, Università del Piemonte Orientale (UPO), Corso Trieste 15/A, 28100 Novara, Italy; marta.mellai@uniupo.it (M.M.); marta.allesina@uniupo.it (M.A.); 4 Department of Chemistry “Ugo Schiff”, University of Florence, Via della Lastruccia 3-13, Sesto Fiorentino, 50019 Florence, Italy; alessia.vignoli@unifi.it (A.V.); leonardo.tenori@unifi.it (L.T.); 5 Magnetic Resonance Center (CERM), University of Florence, Via Luigi Sacconi 6, Sesto Fiorentino, 50019 Florence, Italy; 6 Odontostomatology Unit, Azienda Ospedaliero Universitaria (AOU) “Maggiore della Carita”, 28100 Novara, Italy; monica.marotta@maggioreosp.novara.it (M.M.); mario.migliario@med.uniupo.it (M.M.); 7 Biological Mass Spectrometry, Center for Translational Research on Allergic and Autoimmune Diseases (CAAD), Department of Translational Medicine (DiMeT), School of Medicine, Università del Piemonte Orientale (UPO), Corso Trieste 15/A, 28100 Novara, Italy; marcello.manfredi@uniupo.it; 8 Center for Translational Research on Allergic and Autoimmune Diseases (CAAD), Department of Sciences and Technological Innovation (DISIT), Università del Piemonte Orientale (UPO), 15121 Alessandria, Italy; elettra.barberis@uniupo.it; 9 Department of Translational Medicine (DiMeT), School of Medicine, Università del Piemonte Orientale (UPO), 28100 Novara, Italy; 10 Protein Technologies Core Facility, Center for Translational Research on Allergic and Autoimmune Diseases (CAAD), Department of Translational Medicine (DiMeT), School of Medicine, Università del Piemonte Orientale (UPO), Corso Trieste 15/A, 28100 Novara, Italy; valeria.caneparo@med.uniupo.it; 11 Probiotical Research S.R.L., Via Mattei 3, 28100 Novara, Italy; a.amoruso@probiotical.com (A.A.); m.pane@probiotical.com (M.P.)

**Keywords:** oral lichen planus (OLP), oral and gut microbiota, vitamin D, probiotics, cytokines, metabolomics, proteomics, 16S RNA sequencing

## Abstract

Oral lichen planus (OLP) is a chronic inflammatory oral disease associated with immune dysregulation and malignant transformation risk. Vitamin D and probiotics may modulate immune and microbial pathways involved in OLP. In this study, we evaluated their effects on clinical outcomes and multi-omics profiles in 25 adult OLP patients (median age: 68 years). Vitamin D-deficient patients received 2000 IU/day vitamin D3, and all participants received a probiotic blend (*Limosilactobacillus reuteri* LRE11, *Lacticaseibacillus rhamnosus* LR04, and *Lacticaseibacillus casei* LC04) for 16 weeks. Clinical assessments and analyses of saliva, serum, oral swabs, and stool samples were performed before and after treatment. Clinical outcomes improved significantly, with reductions in lesion number (*p* < 0.001), lesion size (*p* = 0.004), and bleeding lesions (*p* = 0.014); 76% of patients were classified as in remission. Vitamin D levels increased significantly among deficient patients receiving correction (*p* < 0.01). Salivary and fecal metabolomics showed significant remodeling of amino acid and carbohydrate pathways, including decreases in branched-chain amino acids in saliva and modulation of nicotinamide- and amino acid-related metabolites in feces. Microbiome α-diversity remained stable, whereas β-diversity shifted significantly across oral and fecal sites, with enrichment of *Lacticaseibacillus* and other context-dependent commensals. Multi-omics integration identified three latent factors linking salivary cytokines, microbial taxa, metabolites, and systemic lipid profiles, suggesting coordinated mucosal–metabolic–immune remodeling across the oral–gut axis.

## 1. Introduction

Oral lichen planus (OLP) is a chronic, T-cell-mediated inflammatory disorder affecting the oral mucosa. It has a global prevalence of approximately 0.5–4%, with a higher occurrence in females [1]. Despite its known risks, OLP remains underdiagnosed or misdiagnosed due to the lack of standardized criteria [2,3,4]. Its variable clinical presentation, which may be asymptomatic or mimic other oral conditions, combined with limited awareness among patients and non-specialist healthcare providers, further complicates its recognition [5]. In classical lesions (bilateral, reticular pattern), it is possible to make a diagnosis based on the clinical appearance alone even if a definitive diagnosis requires histological examination, which is not always performed because it is not invariably accepted by asymptomatic patients [6,7].

OLP onset and progression are influenced by multiple factors, including genetic predisposition, trauma, and environmental stressors, such as dietary components and chemicals. These factors can disrupt oral and gut microbiota, leading to dysbiosis. The depletion of beneficial microbial species allows opportunistic pathogens to flourish, contributing to chronic inflammation and, potentially, to carcinogenesis [8,9,10,11,12].

A hallmark of OLP is a dense, band-like lymphocytic infiltrate at the epithelial-basal membrane interface, resulting in basal cell degeneration and epithelial destruction [13]. The immune response involves macrophages, monocytes, CD4+ and CD8+ T cells, dendritic cells, fibroblasts, and epithelial cells, which together drive cytokine overproduction, nuclear factor-kappa B (NF-κB) signaling activation and basal epithelial cell apoptosis. Matrix metalloproteinases (MMPs) and mast cell degranulation further sustain inflammation and tissue damage [14,15].

Clinically, OLP manifests in six forms (i.e., reticular, atrophic, papular, bullous, plaque-like, and erosive-ulcerative), which may coexist in the same patient and may involve extraoral sites, significantly impacting the patient’s life quality [13,16]. Although its malignant potential was discovered over a century ago, the World Health Organization (WHO) only officially acknowledged the risk in 2005, with an estimated transformation to oral squamous cell carcinoma (OSCC) of 0.44–5%, with higher incidence in erosive/ulcerative forms, tongue lesions, female sex, and the 6th to 7th decade of life, but none of these have achieved significant agreement among researchers [17,18,19,20].

Current OLP treatments (corticosteroids, retinoids, and immunosuppressants such as tacrolimus) primarily aim to control inflammation and symptoms. However, concerns regarding their long-term safety and variable efficacy have prompted the search for alternative solutions [21,22,23].

Recent research highlights the role of gut microbiota dysbiosis in OLP, particularly its impact on regulatory T cell (Treg) function [24]. This has spurred interest in probiotics as a potential adjunctive therapy. Specific strains, such as *Lacticaseibacillus rhamnosus*, *Lactobacillus delbrueckii*, *Lactobacillus johnsonii*, *Lactobacillus plantarum*, and *Bifidobacterium animalis*, exhibit immunomodulatory properties, influencing the Treg/T-helper 17 (Th17) axis, suppressing excessive immune responses, and promoting immune homeostasis [25,26,27]. Probiotics can also modulate inflammatory cytokines, such as interleukin (IL)-10 and transforming growth factor-beta (TGF-β), thereby mitigating chronic OLP-associated inflammation. Evidence from our group demonstrated that probiotic Cell Free Supernatants (CFSs), together with vitamin D, reduce IL-6 production and counteract cytotoxicity in oral epithelial cells exposed to periodontal pathogens [28].

In addition, other studies on other immune-mediated diseases such as rheumatoid arthritis [29] and experimental autoimmune encephalomyelitis [30] suggest that probiotics enhance Treg differentiation, modulate cytokine profiles, and inhibit NF-κB signaling, providing a rationale for their application in OLP.

Vitamin D, a known immunomodulator, can suppress pro-inflammatory T-helper 1 (Th1)/Th17 responses, while enhancing regulatory cytokines [31]. Since vitamin D deficiency has been frequently reported in OLP and has been associated with higher disease activity, correcting low vitamin D status may contribute to symptom/lesion improvement and to the modulation of inflammatory readouts, including interferon-gamma (IFN-γ) [32].

Based on these premises, probiotic supplementation and vitamin D correction may act as complementary immuno-microbiome modulators, potentially promoting mucosal immune homeostasis and microbial compositional shifts, while systemic effects remain to be clarified [33,34]. Despite promising preliminary evidence, several uncertainties continue to limit the clinical application of probiotics in OLP management. These include the unknown precise mechanisms of immune modulation, the identification of optimal strains, appropriate dosage regimens and delivery methods, variability in therapeutic response across OLP subtypes, potential interactions with conventional treatments, absence of validated predictive biomarkers, and limited long-term safety data.

This exploratory pilot monocentric study investigates the impact of a 16-week intervention combining vitamin D correction (according to baseline status) and multi-strain probiotic supplementation on clinical outcomes and multi-layer profiling in 25 OLP patients, encompassing saliva and serum cytokines, serum lipoprotein measures, metabolomics profiles (saliva, serum, and stool), and 16S rRNA-based microbiome data (saliva, stool, and lesional/non-lesional oral sites).

We aim to determine whether the intervention can improve clinical symptoms, modulate inflammatory responses, induce metabolic adaptations, and reshape microbial community composition. Findings from this study will inform larger trials to validate these effects, elucidate mechanisms of vitamin D- and probiotic-mediated immune regulation, and support the development of microbiota-based strategies for personalized OLP management.

## 2. Results

### 2.1. OLP Patients’ Population

A total of 25 patients were enrolled, of whom 68% were female. The median age was 68 years (IQR: 58–73). The median BMI was 24.0 kg/m^2^ (IQR: 22.5–29.1), with 60% of participants classified as normal weight, 20% as overweight, and 20% as obese (Table 1).

**Figure 1:**
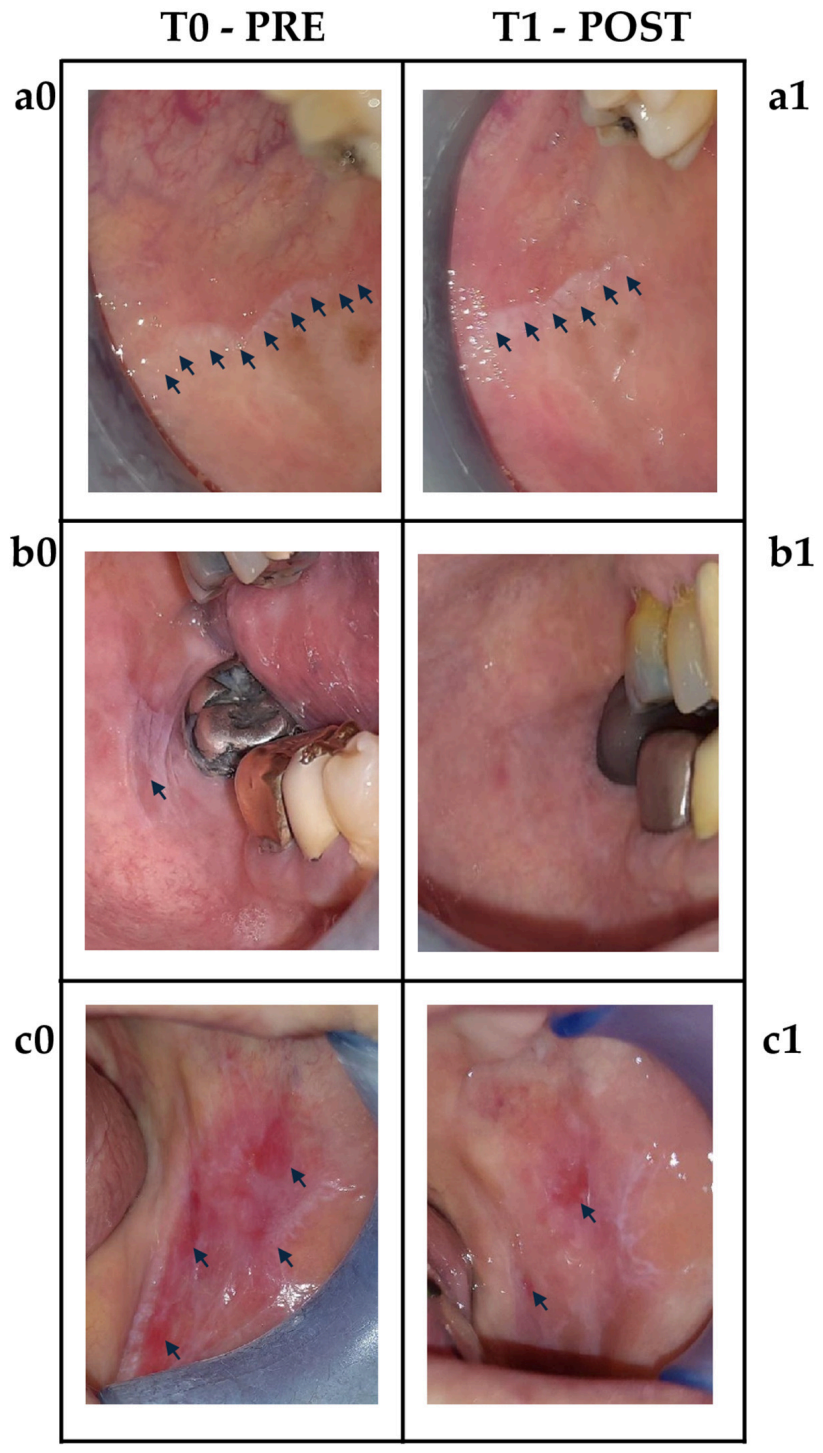
OLP patient’s lesions at enrollment (PRE, **left**) and after 16 weeks of therapy administration (POST, **right**). Representative photographs of mild (**a0**,**a1**), mild to severe (**b0**,**b1**), and severe (**c0**,**c1**) OLP cases highlighted with arrows. First case (**a0**): reticular OLP of right cheek, reduced in size after therapy (**a1**). Second case (**b0**): plaque-like OLP of right cheek, resolved after therapy (**b1**). Third case (**c0**): erosive-ulcerative OLP of the left cheek; the number of erosive lesions is reduced after therapy (**c1**).

OLP clinical diagnosis was based on objective examination only in 44% of the patients, while the remaining 56% had biopsy confirmation. Patients were classified at baseline according to disease severity based on lesion type: 36% mild (reticular lesions only), 20% mild-severe (papular/plaque-like or atrophic lesions without erosive/ulcerative lesions), and 44% severe (erosive/ulcerative lesions). Representative lesion-type images are provided in Figure 1.

Lesion distribution across oral sites and clinical patterns in the cohort are summarized in Supplementary Materials Table S1. Cheek involvement was universal, with reticular lesions representing the predominant pattern (20/25, 80%), while papular/plaque-like (3/25, 12%) and erosive (2/25, 4%) were uncommon. Tongue involvement was less frequent, as 17/25 (68%) showed no tongue lesions; when present, tongue lesions were heterogeneous and mainly atrophic (3/25, 12%), with reticular and papular/plaque-like patterns each observed in 2/25 (8%) and erosive lesions in 1/25 (4%). Gingival lesions were observed in just over half of patients (13/25, 52%) and were largely erosive (11/25, 44%, i.e., the 100% of severe cases), whereas papular/plaque-like gingival lesions were rare (2/25, 8%).

Comorbidities and ongoing systemic therapies were collected as part of baseline cohort characterization (Supplementary Materials Tables S2–S5). Potentially OLP-related comorbidities and other comorbidities are summarized in Supplementary Materials Tables S2 and S3, respectively. Hypertension was the most frequent potentially OLP-related comorbidity (15/25, 60%), followed by diabetes (7/25, 28%), while hypothyroidism and psychosis were each observed in 4/25 (16%) patients. Regarding other conditions, osteoporosis was the most prevalent (8/25, 32%), followed by blood diseases, cardiovascular diseases, and solid tumors, which were each reported in 5/25 (20%) individuals. Systemic medications potentially associated with OLP and medications potentially decreasing salivary flow are reported in Supplementary Materials Tables S4 and S5. Anti-hypertensives represented the predominant therapy in both drug categories (15/25, 60%). Other medications potentially associated with OLP included oral hypoglycemics (5/25, 20%), whereas the remaining treatments linked to decreased salivary flow were mainly antacids (5/25, 20%), psychiatric drugs (4/25, 16%), and diuretics (3/25, 12%).

### 2.2. Vitamin D Correction

A summary of vitamin D correction types and serum concentrations at the baseline is presented in Supplementary Materials Table S6. Vitamin D correction was not required in 10 patients, as their baseline levels were within the normal range (20–40 ng/mL) according to the Italian Drug Agency (Agenzia Italiana del Farmaco, AIFA). Of the remaining patients, 8 were already receiving pharmacological supplementation (Dibase, Didrogyl), while 7 started vitamin D correction at PRE.

**Figure 2:**
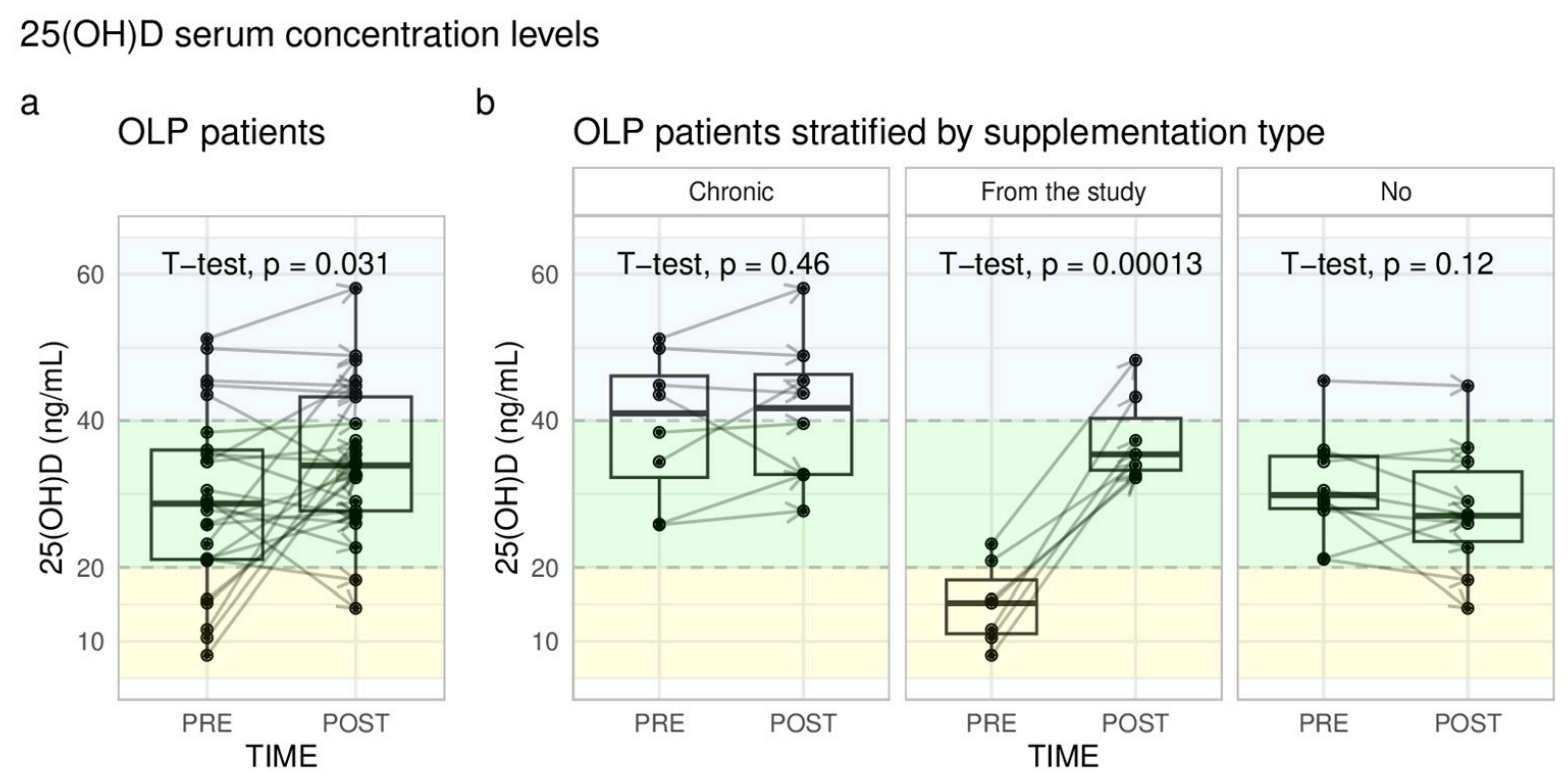
Vitamin D levels in OLP patients at enrollment (PRE) and after 16 weeks of correction (POST). Serum 25[OH]D levels measured at PRE and POST, expressed in ng/mL. Concentration ranges are highlighted with different colors according to the Italian Drug Agency (AIFA): deficiency in yellow (<20 ng/mL), normal range in green (20–40 ng/mL), high values in blue (40–100 ng/mL). (**a**) All OLP patients. T-test for paired samples showed significant concentration differences between time points (*p* < 0.05). (**b**) OLP patients stratified by vitamin D supplementation type: chronic users, correction initiated during the study, and no correction. T-test for paired samples showed significant concentration differences between time points for patients who initiated correction during the study (*p* < 0.05).

Vitamin D levels in patients’ sera were also measured at POST. Overall, a significant increase (*p* = 0.03) was observed from PRE to POST (Figure 2a). More specifically, patients who had vitamin D levels within the normal range at PRE, whether chronically supplementing or not, showed an almost stable trend over time (Figure 2b). In contrast, those who started vitamin D correction for the first time at enrollment exhibited a significant increase in 25[OH]D levels (*p* < 0.01).

### 2.3. Clinical Response

**Figure 3:**
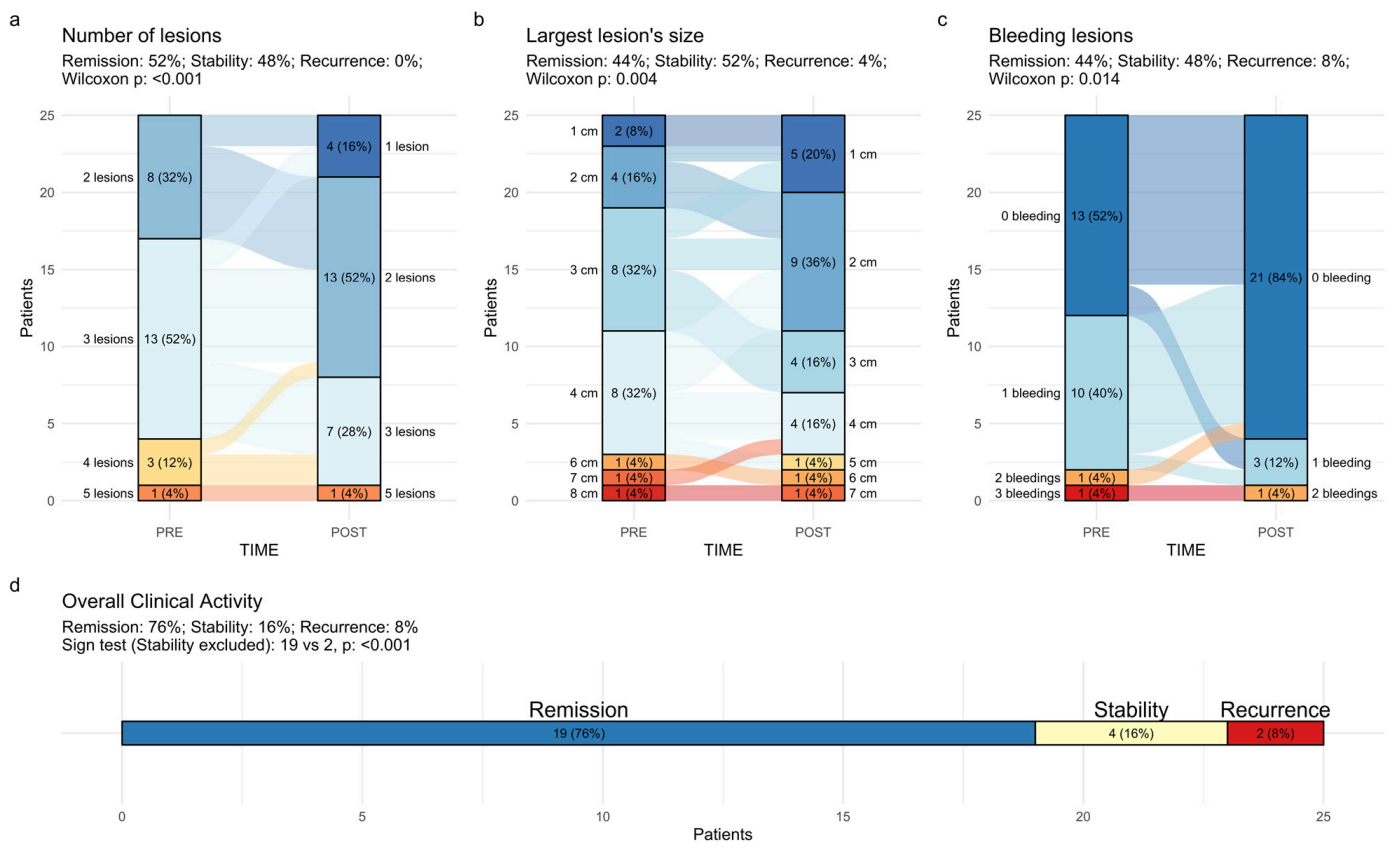
Individual changes in lesion-related outcomes and overall clinical activity after the 16-week intervention period. Panels (**a**–**c**) display patient-level transitions from baseline (PRE) to post-treatment (POST) for (**a**) lesion number, (**b**) largest lesion size (cm), and (**c**) number of bleeding lesions, with strata indicating the distribution across categories at each time point. Paired PRE-POST differences were assessed using the Wilcoxon signed-rank test. Panel (**d**) shows the composite clinical activity classification (see Section 4). The directional imbalance toward remission was evaluated using a sign test excluding stable cases.

Lesion characteristics improved from baseline to post-intervention (Figure 3). Lesion number showed a statistically significant change (*p* < 0.001), with most patients shifting toward lower lesion counts (52%, Figure 3a). Largest lesion size also changed significantly (*p* = 0.004), with an overall redistribution toward smaller size categories (Figure 3b). Bleeding activity improved (*p* = 0.014), with a higher proportion of patients showing no bleeding lesions at POST (52% vs. 84%, Figure 3c). To integrate changes across lesion number, largest lesion size, and bleeding lesions, a composite Clinical Activity Index was derived by assigning each marker a score (−1 remission, 0 stability, +1 recurrence) and summing scores across markers; 19 patients were classified as overall remission (76%), with 4 remaining stable (16%) and only 2 showing recurrence (8%). The distribution was strongly skewed toward remission (*p* < 0.001; Figure 3d).

### 2.4. Inflammatory Profile

**Figure 4:**
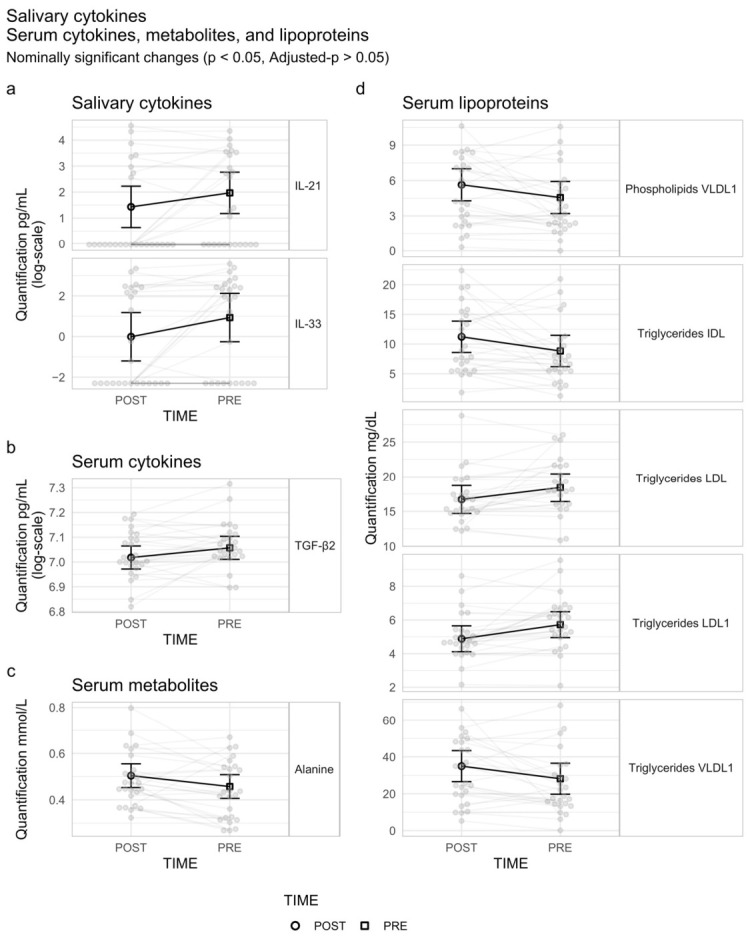
Adjusted quantifications before (PRE) and after (POST) the 16-week intervention. Values represent estimated marginal means (and 95% CI) obtained from linear mixed-effects models adjusted for baseline covariates (age, sex, BMI, baseline vitamin D concentration, and disease severity). Only nominally significant changes (*p* < 0.05) are shown; no adjusted *p*-values remained significant after multiple-testing correction. (**a**) Salivary cytokines. (**b**) Serum cytokines. (**c**) Serum metabolites. (**d**) Serum lipoproteins.

To explore whether the intervention was associated with immune modulation, we evaluated time-related changes across a panel of salivary and serum cytokines. After multiple-testing correction, no cytokine met the significance threshold (*p* = 0.05); nevertheless, the largest percentage reductions were observed for salivary IL-21 (42%) and IL-33 (61%), with a small decrease in serum TGF-β2 (4%) (Figure 4a,b, Table 2 and Table 3).

### 2.5. Serum Metabolomics and Lipoproteomics Analyses

Following the cytokine analyses, we next assessed whether the intervention was associated with broader systemic biochemical changes by examining the serum NMR metabolomics and lipoprotein profiles.

After FDR correction, no serum metabolite or lipoprotein feature reached statistical significance. Descriptively, the most notable absolute changes were an increase in alanine and shifts in lipoprotein measures, including higher phospholipids in VLDL1 and higher triglycerides in IDL and VLDL1, alongside lower triglycerides in LDL and LDL1 (Figure 4c,d, Supplementary Materials Tables S7 and S8).

### 2.6. Salivary and Fecal Metabolomics Analyses

We then extended profiling to untargeted GC×GC–MS metabolomics in saliva and feces to capture local and intestinal metabolic readouts potentially accompanying the intervention.

Untargeted metabolomics analysis detected and quantified 330 salivary and 255 fecal metabolites. After multiple testing corrections, 85 salivary metabolites (16 increased, 69 decreased; Supplementary Materials Table S9) and 34 fecal metabolites (19 increased, 15 decreased; Supplementary Materials Table S10) changed significantly (adjusted *p* < 0.05).

In saliva, significant changes included decreases in L-threonine and other amino acids (e.g., L-leucine, L-valine), together with decreases in carbohydrate-related features (β-D-(+)-xylopyranose, D-arabinose, D-mannose, ribitol, 1,5-anhydroglucitol) and in tryptophan-related metabolites (indoleacetic acid and anthranilic acid) (Supplementary Materials Table S11). Lipid-related features changed in both directions, with decreases in hexadecanamide, octadecanamide, and palmitic acid and increases in several fatty acids (including myristic acid), while the strongest increases included malonic acid, diglycerol, xylitol, L-aspartic acid, and hippuric acid (Supplementary Materials Table S9).

In feces, significant changes included decreases in pimelic acid, glycine, D-(−)-fructofuranose, and 3-hydroxybutyric acid (β-hydroxybutyrate), alongside increases in methyl α-D-glucofuranoside, catechol, 1-monooleoylglycerol, xylonic acid, erythritol, niacinamide, adenine, and the dipeptide Ser–Leu (Supplementary Materials Table S10).

**Figure 5:**
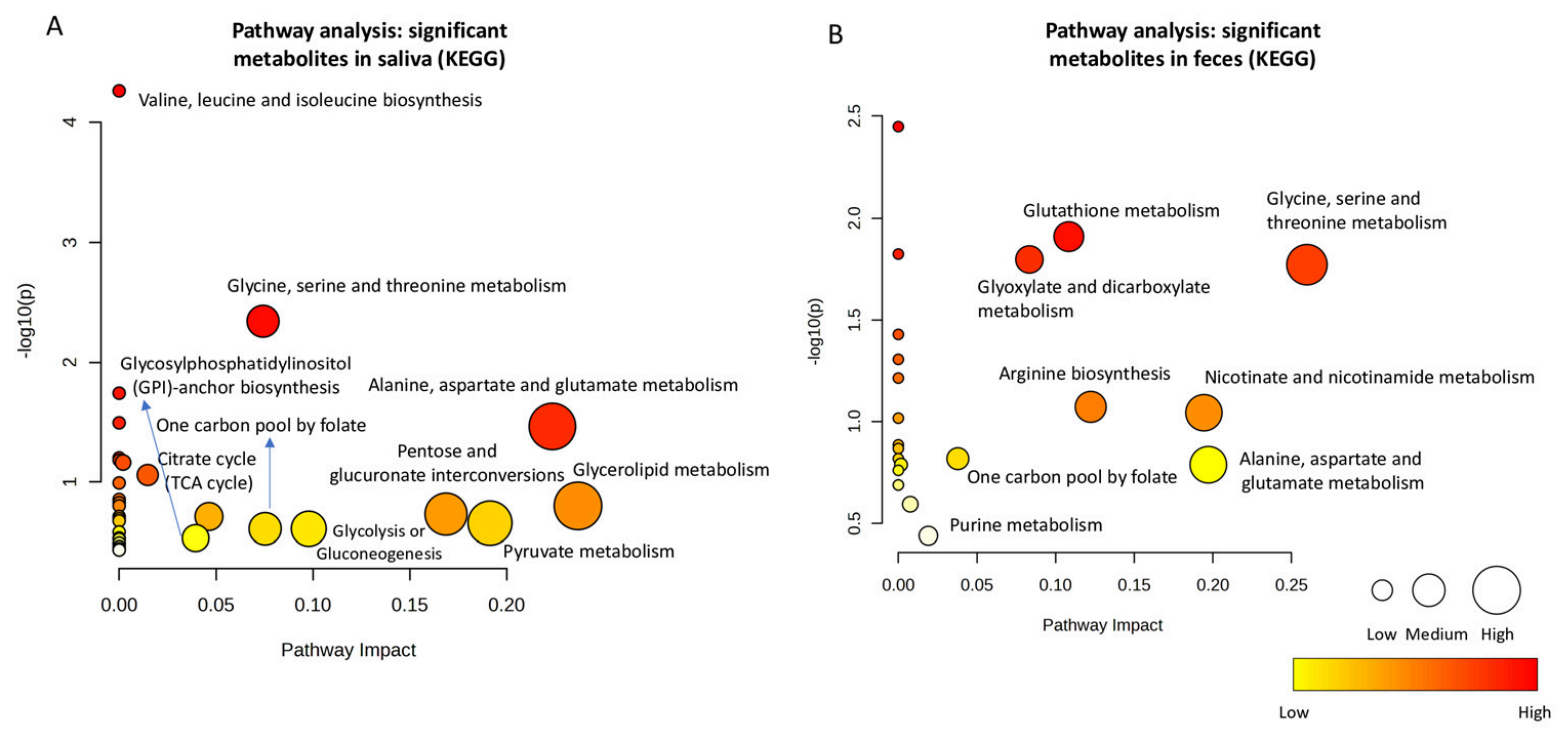
KEGG pathway impact plots of significantly changed metabolites in saliva (**A**) and feces (**B**) following the 16-week intervention. Pathways were identified using over-representation analysis with pathway topology impact. The *y*-axis shows enrichment significance (−log10 *p*-value), and the x-axis shows pathway impact from topology analysis; larger and darker bubbles indicate pathways with greater impact and significance. Selected pathways with the highest impact and/or significance are labeled in each panel.

Pathway analysis highlighted valine, leucine and isoleucine biosynthesis and alanine, aspartate and glutamate metabolism in saliva (Figure 5A), and glycine, serine and threonine metabolism together with alanine, aspartate and glutamate metabolism in feces (Figure 5B).

### 2.7. 16S rRNA Gene Sequencing

To complement the immune and metabolomics layers, we next profiled the oral and gut microbiomes using 16S rRNA gene metabarcoding across S, L, NL, and F samples, focusing on community structure (α/β-diversity) and genus-level differential abundance between time points.

#### 2.7.1. Microbiome Composition

Sequencing generated high-quality 16S rRNA gene data across all sample types (S, L, NL, and F; Supplementary Materials Table S11). All samples exceed 20,000 high-quality reads. Rarefaction curves (Supplementary Materials Figure S1) approached saturation, indicating that microbial richness was adequately captured in all samples.

**Figure 6:**
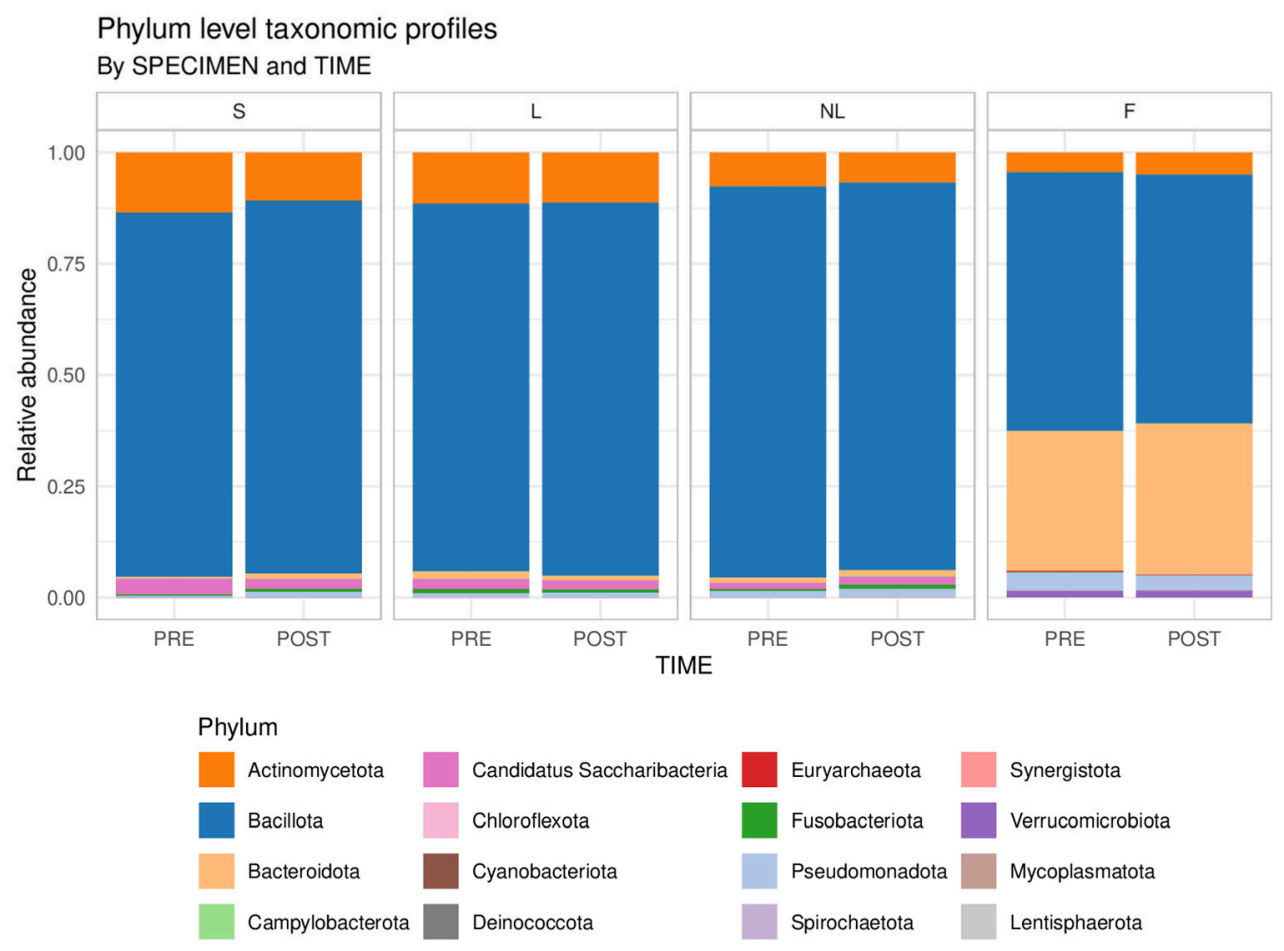
Phylum-level average relative abundance profiles in saliva (S), OLP-lesional mucosa (L), non-lesional mucosa (NL), and feces (F) specimens, before (PRE) and after the 16-week intervention period (POST).

After genus-level agglomeration and prevalence filtering, we obtained an average of ~120 microbial features in oral cavity specimens (S, L, and NL) and ~160 features in stool samples (F; Supplementary Materials Table S12). Relative-abundance profiles (Figure 6) showed that the microbial community was overall dominated by members of the Bacillota phylum across all specimen types, with Actinomycetota also contributing substantially. In stool, Bacteroidota represented an additional major component of the community. At the genus level, the dataset encompassed most of the genera belonging to Bacillota, Pseudomonadota, Actinomycetota, and Bacteroidota, while fewer than ten genera were detected for each of the remaining phyla represented in the dataset (see Supplementary Materials Tables S13 and S14 for more details).

#### 2.7.2. Ecological Analyses

**Figure 7:**
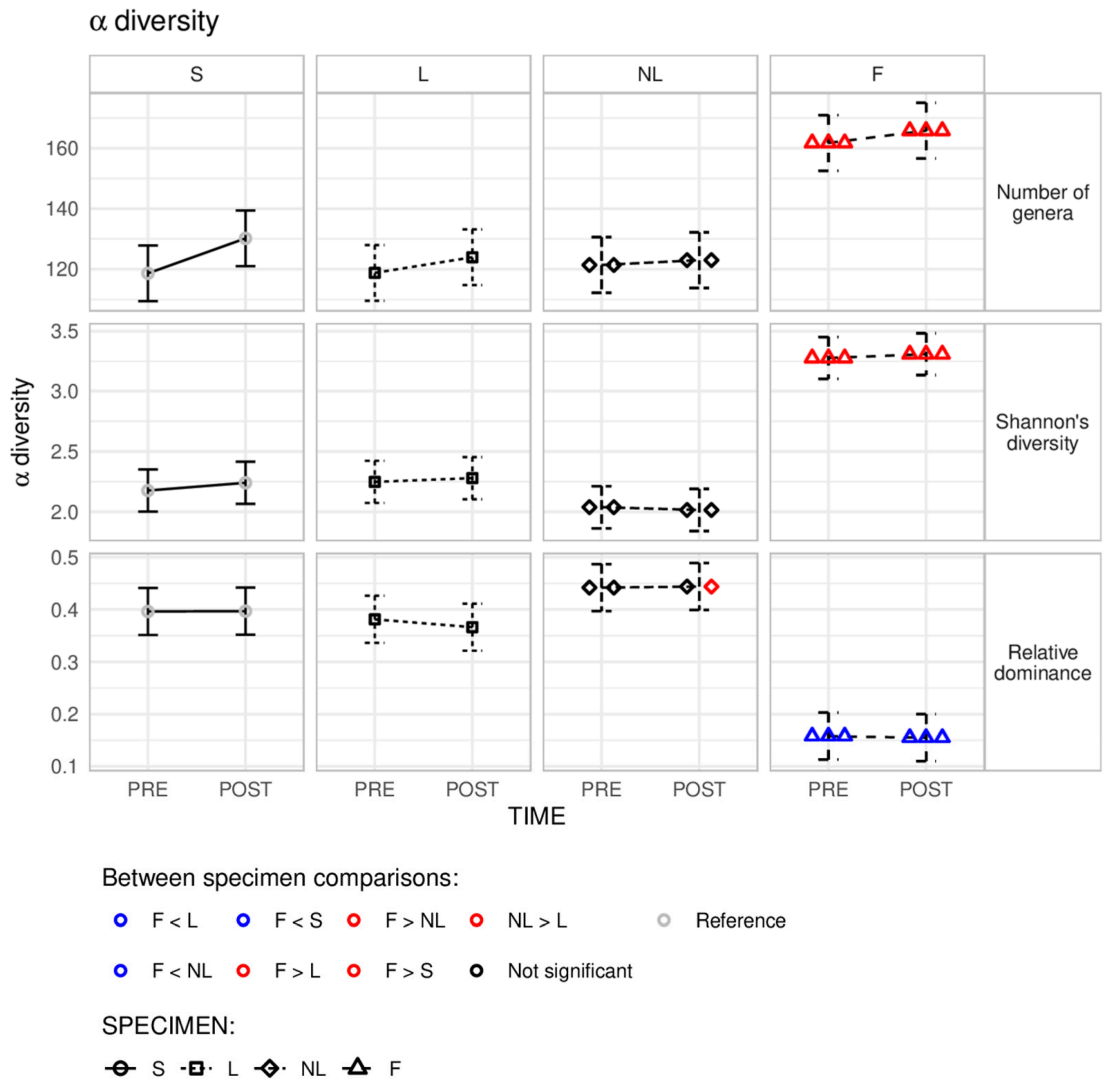
Adjusted α-diversity metrics (number of genera, relative dominance of the most abundant genus, and Shannon diversity index) before (PRE) and after (POST) the 16-week intervention, stratified by specimen type: saliva (S), OLP-lesional mucosa (L), non-lesional mucosa (NL), and feces (F). Values represent model-estimated means (and 95% CI) obtained from linear mixed-effects models adjusted for baseline covariates (age, sex, BMI, baseline vitamin D concentration, and disease severity). Outline colors mark significant differences across specimens (S vs. L, S vs. NL, L vs. NL, S vs. F, L vs. F, NL vs. F) at a given time point. Asterisks indicate significant changes across time points, within each specimen (none are present).

α-diversity metrics showed marked differences between specimen types, whereas no relevant changes were observed over time (Figure 7). Stool samples displayed substantially higher richness and Shannon diversity than all oral cavity specimens (S, L, and NL), with consistently significant contrasts across all metrics (Supplementary Materials Table S15). Oral samples, by contrast, exhibited lower richness and a more uneven taxonomic structure, largely driven by the dominance of a single genus, *Streptococcus*, which accounted on average for approximately 38% of total counts. Relative dominance was therefore significantly higher in oral specimens compared with fecal samples.

Across all specimen types, PRE–POST contrasts showed no significant differences after the 16-week intervention, indicating that α-diversity remained stable over time.

**Figure 8:**
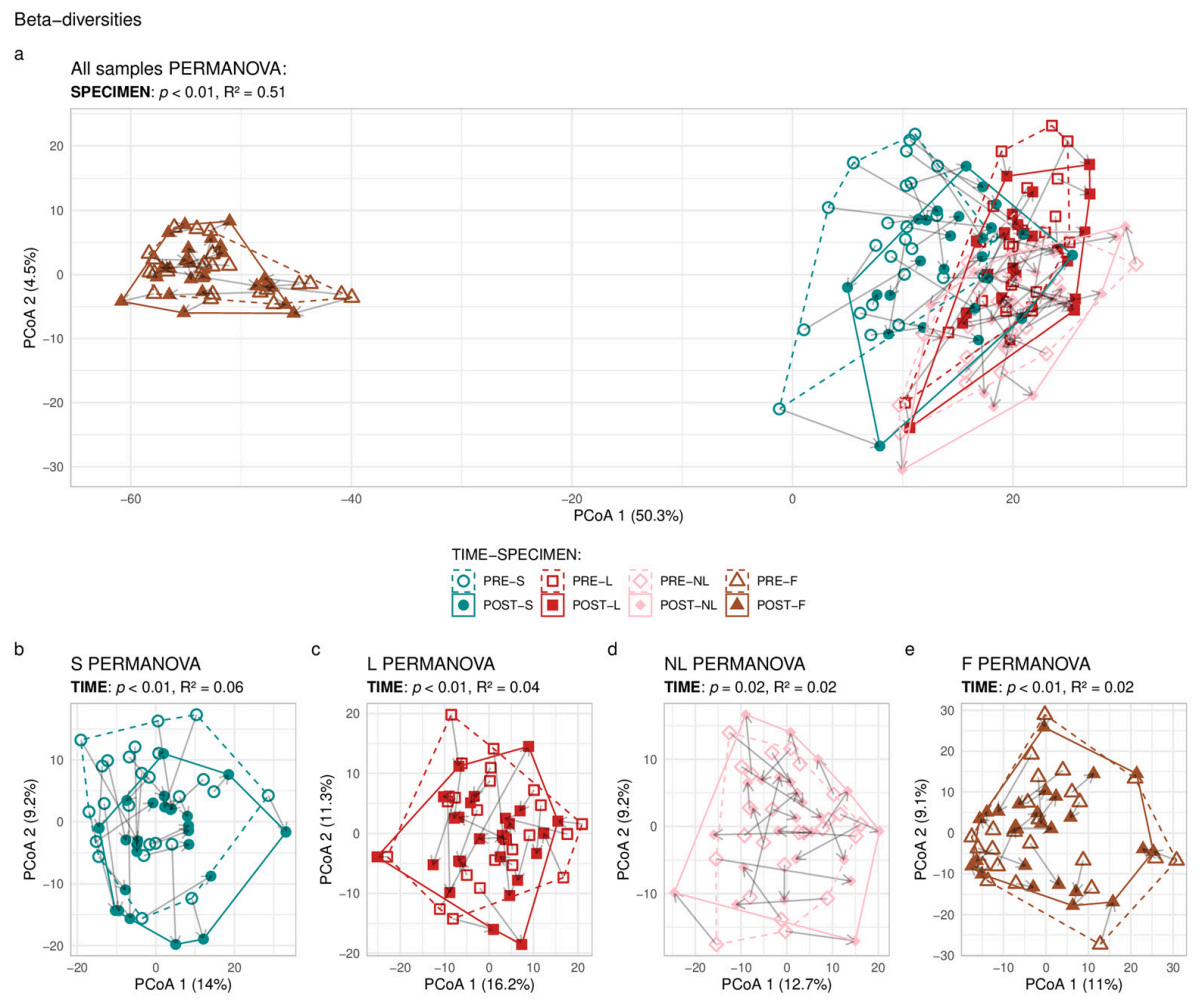
β-diversity based on PCoA of Aitchison distances. Saliva (S), OLP-lesional mucosa (L), non-lesional mucosa (NL), and fecal (F) samples are differentiated by colors and shapes. Empty symbols and dashed convex hulls represent baseline samples (PRE), whereas filled symbols and solid convex hulls represent samples collected after the 16-week intervention (POST). PERMANOVA results are reported in each panel’s title, showing the *p*-value and the proportion of variability explained by the variable of interest (R^2^). Panels: (**a**) all specimens. (**b**) saliva (S) samples. (**c**) OLP-lesional mucosa (L) samples. (**d**) non-lesional mucosa (NL) samples. (**e**) fecal (F) samples.

β-diversity analyses revealed clear global differences in microbial community composition across specimen types. PERMANOVA confirmed that body site accounted for a substantial proportion of overall variability (51.18%, *p* < 0.01), with significant between-specimen dissimilarities and heterogeneous dispersion patterns, particularly lower dispersion in fecal samples (*p* < 0.01, Figure 8a). When analyzing each body site separately, a significant global effect of the treatment period was detected (*p* < 0.01 in S, L, F, and *p* = 0.02 in NL), indicating that the intervention was associated with shifts in microbial composition across all specimen types (Figure 8b–e).

#### 2.7.3. Differential Abundance Analysis

**Figure 9:**
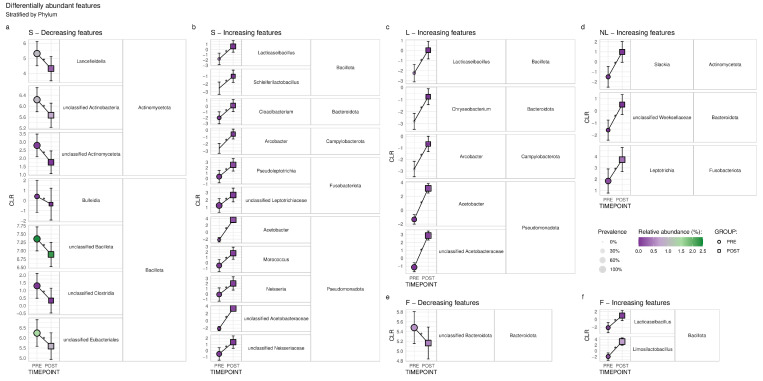
Differentially abundant taxa before (PRE, circle-shaped) and after (POST, square-shaped) the 16-week intervention. Values represent model-estimated means obtained from linear mixed-effects models adjusted for baseline covariates (age, sex, BMI, baseline vitamin D concentration, and disease severity). Point size reflects prevalence across samples, and point color represents relative abundance. Asterisks indicate significant changes across time points. (**a**) Saliva (S) decreased taxa. (**b**) Saliva (S) increased taxa. (**c**) OLP-lesional mucosa (L) increased taxa. (**d**) Non-lesional mucosa (NL) increased taxa. (**e**) Feces (F) decreased taxon. (**f**) Feces (F) increased taxa.

The differential abundance analysis identified several taxa whose CLR abundances differed significantly between PRE and POST (Figure 9). Patterns were highly specimen-specific.

In saliva, several genera within Actinomycetota (e.g., *Lancefieldella*) and Bacillota (e.g., *Bulleidia*) phyla showed decreased abundances at POST (Figure 9a). In contrast, significant increases were observed for *Lacticaseibacillus* and *Schleiferilactobacillus* (Bacillota), *Cloacibacterium* (Bacteroidota), *Arcobacter* (Campylobacterota), *Pseudoleptotrichia*, *Acetobacter*, *Morococcus*, and *Neisseria* (Pseudomonadota) (Figure 9b). A small number of additional unclassified taxa also displayed changes in the same directions.

OLP-lesional mucosa samples showed a clear tendency toward increased abundances at POST (Figure 9c). Significant increases were detected in genera belonging to Bacillota, Bacteroidota, Campylobacterota, and Pseudomonadota, including *Lacticaseibacillus*, *Chryseobacterium*, *Arcobacter*, and *Acetobacter*, respectively. A few additional unclassified taxa also exhibited increases.

Non-lesional mucosa also showed exclusively increased taxa at POST (Figure 9d), including genera from Actinomycetota, Bacteroidota, and Fusobacteriota (e.g., *Slackia*, unclassified *Weeksellaceae*, and *Leptotrichia*), with no significant decreases detected.

Stool samples exhibited fewer changes overall (Figure 9e,f). Unclassified Bacteroidota sequences decreased, while two Bacillota genera, *Lacticaseibacillus* and *Limisilactobacillus*, showed increases, consistent with the composition of the administered probiotic.

### 2.8. Multi-Omics Integration

To identify coordinated time-related changes shared across molecular layers, we applied MOFA to covariate-adjusted within-subject deltas (POST-PRE) from cytokines, metabolomics, serum lipoproteins, and microbiome profiles. The final model retained three latent factors, here called “Factors”, summarizing the major axes of cross-omics covariation.

**Figure 10:**
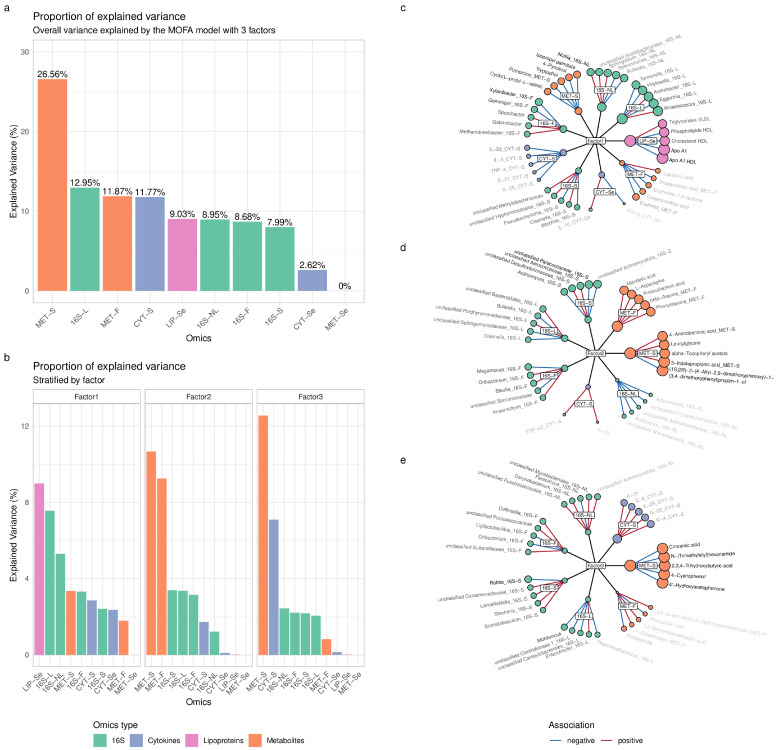
Multi-omics integration and latent factor structure identified by MOFA. The MOFA model integrates microbiome profiles from saliva, lesional and non-lesional oral mucosa, and feces (16S-S, 16S-L, 16S-NL, 16S-F), salivary and serum cytokines (CYT-S, CYT-Se), salivary and fecal metabolomics (MET-S, MET-F), as well as serum metabolites and lipoproteins (MET-Se, LIP-Se). (**a**) Proportion of variance explained within each omics layer (i.e., view) by the full MOFA model, indicating the extent to which latent factors capture variability within each view. (**b**) Variance explained (R^2^) within each omics layer stratified by latent factor, showing how much variability within each molecular layer is captured by each latent factor. (**c**–**e**) Top-weighted features associated with Factor 1 (**c**), Factor 2 (**d**) and Factor 3 (**e**). For each factor, the five features with the highest absolute weights (|weight| > 0.1) were selected independently within each omics layer. Feature-factor edges are colored according to the sign of the weight, indicating positive or negative association with the corresponding factor. Feature node size is proportional to the absolute value of the weight, while label transparency further reflects feature magnitude. Omics layer node sizes are proportional to the variance explained (R^2^) by the corresponding factor, providing a visual summary of factor-specific variance distribution across views.

Across the model, salivary metabolomics showed the largest cumulative fraction of variance explained (26.56%), followed by 16S-L (12.95%), MET-F (11.87%), CYT-S (11.77%), and LIP-Se (9.03%). Smaller contributions were observed for 16S-NL (8.95%), 16S-F (8.68%), 16S-S (7.99%), and CYT-Se (2.62%), while MET-Se contributed negligibly (Figure 10a). When stratified by factor, Factor 1 displayed broad multi-view contributions (notably lipoproteins and several microbiome compartments), Factor 2 was predominantly characterized by metabolomics, and Factor 3 by salivary metabolomics and salivary cytokines (Figure 10b). To facilitate interpretation, each factor was examined individually by considering view-level variance contributions and top-weighted features (|weight| > 0.1).

Factor 1 captured coordinated variance across serum lipoproteins, oral and gut microbiome profiles (mainly 16S-L and 16S-NL), and salivary cytokines (Figure 10b). Higher Factor 1 scores were associated with negative weights for HDL-related components (Apo A1, Apo A1/HDL, HDL cholesterol, HDL phospholipids) and positive weights for VLDL triglycerides. Microbial weights were distributed across oral and fecal genera with mixed directions across compartments, while salivary cytokines (including IL-33, IL-4, TNF-α, IL-21, and IL-25) consistently showed negative weights (Figure 10c). Overall, Factor 1 delineates a coordinated axis linking systemic lipid remodeling with microbial variation across oral and fecal niches, accompanied by inverse shifts in salivary cytokines.

Factor 2 was primarily driven by metabolomics (MET-S and MET-F), with smaller contributions from microbiome views and limited involvement of salivary cytokines (Figure 10b). Higher Factor 2 scores were characterized by positive weights for salivary metabolites such as indole-3-propionic acid, leucylglycine, and 4-aminobenzoic acid and negative weights for α-tocopheryl acetate. Fecal metabolomics also contributed positively through amino acids including phenylalanine, β-alanine, and L-asparagine (Figure 10d). Microbial contributions spanned oral and fecal genera (e.g., *Actinomyces*, *Olsenella*, *Bulleidia*, *Blautia*, *Anaerostipes*), while salivary cytokine involvement was limited and positive (TGF-β2 and IL-23). Collectively, Factor 2 represents a metabolite-dominant axis with partial microbial and limited cytokine contributions.

Factor 3 was mainly supported by salivary metabolomics (MET-S) and salivary cytokines (CYT-S), with smaller contributions from microbiome views and minimal input from fecal metabolomics (Figure 10b). Higher Factor 3 scores were characterized by consistently positive weights for salivary cytokines, including IL-4, IL-33, IL-25, IL-6, and IL-31. In contrast, the top-weighted salivary metabolites (4′-hydroxyacetophenone, 4-cyanophenol, 2,3,4-trihydroxybutyric acid, N-(trimethylsilyl) hexanamide, cinnamic acid) displayed negative weights (Figure 10e). Microbial weights were distributed across oral and fecal genera without a single dominant compartment. Overall, Factor 3 delineates a localized salivary inflammatory axis characterized by coordinated cytokine increases and inverse shifts in specific salivary metabolites.

Phylum-level average relative abundance profiles in saliva (S), OLP-lesional mucosa (L), non-lesional mucosa (NL), and feces (F) specimens before (PRE) and after the 16-week intervention period (POST).

## 3. Discussion

OLP is a complex inflammatory immune-based disorder with an unclear etiology and an estimated risk of transformation to oral squamous cell carcinoma (OSCC) up to about 5%. Therapeutic options remain limited, although increasing evidence suggests a potential role for adjunctive vitamin D correction and probiotic-based interventions. Vitamin D deficiency has been implicated in OLP pathogenesis, and its supplementation has been reported to exert immunomodulatory effects, including modulation of pro-inflammatory cascades and epithelial apoptosis [35,36].

Similarly, probiotic supplementation in OLP has yielded heterogeneous results, likely reflecting strain-specific effects, dosage regimens, treatment duration, and population variability. Some studies have reported clinical improvements with specific strains, such as *Streptococcus salivarius* K12, whereas others have not demonstrated consistent effects with *Lactobacillus reuteri*-based formulations [21,37,38]. Combined vitamin D and probiotic supplementation strategies have recently been explored, although current evidence remains limited and inconclusive [39,40].

To address this gap, we previously conducted preclinical studies to support strain selection and biological plausibility, identifying three *Lactobacillus* strains (*L. reuteri* LRE11, *L. rhamnosus* LR04, and *L. casei* LC04) with inhibitory activity against oral pathogens (*Aggregatibacter actinomycetemcomitans*, *Streptococcus mitis*, and *Streptococcus mutans*) and immunomodulatory properties in epithelial cell models [28,41]. Based on these findings, the present exploratory in vivo study evaluated the short-term clinical and multi-omics profiles associated with this probiotic blend combined with vitamin D correction in 25 OLP older patients.

### 3.1. Clinical and Inflammatory Outcomes

The 16-week intervention was associated with improvements in clinical parameters, including reductions in lesion number, size, and bleeding activity, with 76% of patients in clinical remission. Although the uncontrolled design and small sample size prevent causal inference, these findings support a potential link between the intervention and short-term clinical improvement in a cohort characterized by predominantly erosive disease, relevant comorbidities, and systemic medications associated with xerostomia [42].

A significant increase in serum vitamin D levels was observed in patients receiving correction therapy, consistent with its homeostatic restoration. In light of previous associations between low vitamin D levels and OLP severity [43,44,45,46], this change may have contributed to the observed clinical trajectory; however, its independent contribution cannot be determined in the present study.

At the immunological level, no salivary or serum cytokine changes reached statistical significance after multiple-testing correction. Still, consistent directional variations were observed in salivary inflammatory mediators, particularly IL-21 and IL-33, which are involved in Th17-associated and alarmin-related pathways, respectively [47,48]. Such patterns are mechanistically consistent with the anti-inflammatory properties documented in vitro [28] and may reflect modulation of the local inflammatory environment.

### 3.2. Metabolic and Lipid Profiles

As OLP has been positively associated with dyslipidemia [49,50], we investigated the circulating lipid profile of our cohort. No statistically significant changes were observed in systemic parameters, although descriptive trends suggested subtle alterations in lipoprotein distribution. Phospholipids increased in VLDL1, triglycerides increased in both IDL and VLDL1, and triglycerides decreased in LDL and LDL1 fractions. These changes contrast with the limited literature on vitamin D and probiotic supplementation but may reflect the inflammatory milieu and metabolic context of OLP [51,52]. Given the small size of our cohort, these results should be interpreted cautiously, but they suggest a possible link between OLP-associated inflammation and lipoprotein remodeling.

On the other hand, untargeted metabolomic profiling of saliva and feces revealed more pronounced changes at the mucosal level. In saliva, several amino acids and carbohydrate-related metabolites decreased, including branched-chain amino acids and mucin-associated threonine, suggesting altered epithelial turnover and/or microbial substrate utilization [53]. Concomitant changes in tryptophan-derived metabolites point to modifications in host–microbiome co-metabolic pathways potentially relevant to epithelial barrier function and immune regulation [54].

In feces, metabolomic shifts involved amino acid metabolism and nicotinamide-associated pathways, alongside reduced pimelic acid, a precursor of bacterial biotin (vitamin B7) synthesis, and β-hydroxybutyrate, a metabolite implicated in inflammasome regulation [55,56,57,58].

### 3.3. Site-Specific Microbiota Remodeling Across Oral and Gut Compartments

Microbiome analysis confirmed strong site-specific ecological differences, with oral and gut ecosystems showing distinct baseline compositions. α-diversity did not differ significantly between PRE and POST across specimens, although microbial richness showed an upward trend after intervention, particularly in saliva. In contrast, β-diversity shifted significantly over time across all specimen types, while body site remained the main determinant of microbial variability, consistent with ecological compartmentalization.

Differential abundance analysis showed that salivary microbial communities underwent the most evident remodeling after probiotic supplementation. This included reductions in taxa previously associated with dysbiosis (e.g., *Lancefieldella* and *Bulleidia*) [59,60] and increases in genera commonly considered commensal or context-dependent (e.g., *Neisseria*, *Cloacibacterium*, and *Pseudoleptotrichia*) [61,62]. However, these findings should be interpreted with caution, since elevated levels of *Neisseria* have also been found in saliva from erosive OLP patients [63], whereas *Pseudoleptotrichia goodfellowii*, the only member of the *Pseudoleptotrichia* genus, contributes to dental biofilm [64].

Likewise, the significant enrichment of *Arcobacter*, a genus including both commensal and pathogenic species, requires careful interpretation in light of its reported presence in saliva and plaques from OSCC patients [65,66]. It must be noted that many oral bacteria exhibit context-dependent function. They may behave as commensals within an eubiotic microbial network; yet, favored by exposome-driven selection [67], they may contribute to dysbiosis and inflammation, and other pathogenic strains may take over the niche. In such altered ecosystems, these commensal microbes often express virulence not as overt pathogenicity, but as adaptive survival strategies within the microbial network [68].

The mucosal microbiota is of great interest, as dysbiosis may be more marked there than in saliva and more closely associated with disease features [2,69]. In lesional samples, a similar pattern emerged, with enrichment of *Arcobacter*, *Acetobacter*, *Lacticaseibacillus*, and *Chryseobacterium* genera. Non-lesional specimens were also affected, showing increased abundance of taxa from Actinomycetota, Bacteroidota, and Fusobacteriota (e.g., *Slackia*, unclassified Weeksellaceae, and *Leptotrichia*).

Remarkably, the significant enrichment in the *Lacticaseibacillus* genus in saliva and lesional specimens is consistent with the composition of the probiotic formulation, suggesting successful colonization or transient enrichment in the oral cavity. As discussed, their presence could sustain mucosal barrier function and immune modulation, thus creating favorable conditions for microbial restoration [70,71]. Moreover, the absence of significant bacterial depletion in mucosal samples suggests that the intervention may have favored ecological remodeling rather than direct displacement of resident taxa, improving the mucosal network.

Gut microbiota changes were comparatively modest, consistent with the oral administration of the probiotic formulation and the resilience of the intestinal ecosystem [72]. Nevertheless, the increase in *Lacticaseibacillus* and *Limosilactobacillus* genera (Bacillota) may indicate that the probiotic strains survived gastrointestinal passage and transiently enriched the intestinal niche.

Overall, the different modulation of oral and intestinal compartments suggests that the strongest microbial impact occurred at the delivery site.

### 3.4. Multi-Omics Integration and System-Level Interpretation

OLP is a highly complex disease that cannot be fully captured through univariate analyses. Therefore, MOFA provided a useful framework to integrate immune, metabolomic, and metagenomic changes and summarize shared sources of variation across molecular layers.

In practical terms, three latent factors captured coordinated longitudinal shifts, with the strongest contributions coming from salivary metabolomics, lesional microbiota, fecal metabolomics, and salivary cytokines, whereas blood-based cytokines and metabolites contributed less. This pattern suggests a predominantly mucosal-associated systemic response that extends beyond localized oral inflammation and involves the gut-oral-immune axis. However, given inherent methodological constraints and the limited sample size, these latent factors should be interpreted as exploratory representations of covariation rather than stable biological modules or mechanistic pathways.

Factor 1 captured coordinated variation across lipid-related features, salivary cytokines, and microbial profiles across oral and gut compartments, reflecting correlated host–microbiome–metabolite changes potentially associated with inflammatory status and vitamin D levels [51]. Factor 2 was primarily driven by metabolomic signatures from saliva and feces, with partial microbial contributions, reflecting shared metabolic variability across mucosal ecosystems. Lastly, Factor 3 was largely localized to salivary inflammatory and metabolic features, suggesting patterns of local immune–metabolic covariation.

A key insight from MOFA is the distinction between ‘abundance’ and ‘influence.’ Indeed, *Lacticaseibacillus* genus showed a significant increase in abundance at POST according to univariate analysis, but it did not strongly contribute to the latent factors, suggesting that the observed effects cannot be attributed to probiotic abundance alone. Rather, the clinical response appears to reflect a broader multi-layer reorganization involving microbial, metabolic, and immune components. Within this scenario, we could hypothesize that the probiotic blend may act as a keystone modulator, both directly and indirectly, triggering the complex cascade of metabolic reprogramming that extends far beyond their colonization of the gut or oral cavity.

### 3.5. Limitations

Despite the promising findings of the present study, several limitations must be acknowledged.

The small sample size and single-center design inherently constrain generalizability, and the absence of a placebo-controlled arm precludes definitive attribution of the observed improvements solely to the intervention. Indeed, given the fluctuating clinical course of OLP, contributions from spontaneous remission, regression-to-the-mean, and non-specific effects cannot be formally excluded. The PRE-POST within-subject framework mitigates inter-individual variability by anchoring each participant’s trajectory to their own baseline; linear mixed-effects models further adjust for key covariates (age, sex, BMI, vitamin D status, and disease severity), and systematic multiple-testing correction was applied across all high-dimensional molecular readouts. These analytical choices strengthen internal validity but cannot substitute for randomization. Notably, the relatively small sample size may limit model stability and the risk of overfitting in multi-omics integration; the MOFA-derived latent factors should therefore be interpreted as exploratory summaries of cross-layer covariation rather than evidence of causal or mechanistic relationships.

Diagnostic heterogeneity represents an additional consideration: while 56% of participants had histopathological confirmation, the remaining patients were diagnosed on typical clinical grounds, in line with current practice for classical bilateral reticular lesions. Given the recognized overlap between OLP and oral lichenoid lesions, some degree of diagnostic uncertainty cannot be excluded, and future studies should ideally achieve full histopathological assessment.

Treatment heterogeneity is also inherent to the study design: vitamin D correction was administered only to deficient participants, precluding an independent evaluation of probiotic and vitamin D effects. The observed outcomes should therefore be interpreted as reflecting the overall intervention strategy rather than either component in isolation. Similarly, the 16-week follow-up was sufficient to capture short-term responses but does not allow conclusions on remission durability, recurrence rates, or malignant transformation risk; longer follow-up studies are needed.

A further limitation concerns the composite Clinical Activity Index used to summarize individual clinical trajectories. This instrument was developed specifically for the present study by integrating three clinically relevant parameters (i.e., lesion number, largest lesion size, and bleeding activity) into a single summary score and thus provides an integrated assessment of lesion extent and clinical severity. While the index provides a pragmatically useful summary of multidimensional clinical change within this exploratory context, its reproducibility, sensitivity to change, and correlation with other outcomes remain to be validated in independent cohorts.

Finally, the relatively advanced age of the cohort and the high prevalence of comorbidities and concomitant medications may limit external validity. These characteristics are, however, representative of the OLP population encountered in routine clinical practice, and caution should be exercised when extrapolating findings to younger or clinically distinct populations. Larger, multicenter randomized controlled trials are warranted to confirm and extend these hypothesis-generating results.

## 4. Materials and Methods

### 4.1. Ethical Issues

The study protocol, conducted as an exploratory pilot clinical investigation, was approved by the Ethics Committee (Comitato Etico Interaziendale, CEI) of the Azienda Ospedaliero-Universitaria (AOU) Maggiore della Carità (Novara, Italy), serving as an Institutional Review Board (IRB) (Protocol Number 801/CE, Approval Study Number CE 143/21), in accordance with the Declaration of Helsinki and its Edinburgh amendments [73].

This study was conducted as an exploratory pilot clinical investigation and was not registered on ClinicalTrials.gov or any other public registry.

### 4.2. Study Population

Twenty-five adult OLP patients (clinical and histopathological diagnosis) were enrolled at the Odontostomatology Unit of AOU Maggiore della Carità during the May-September period after obtaining their written informed consent and securing agreement from their family doctor. The inclusion criteria were as follows: patient’s age ≥ 18 years. Furthermore, participants were not allowed to receive any topical or systemic OLP-specific treatment, including corticosteroids, calcineurin inhibitors (e.g., tacrolimus), or other immunosuppressive agents and/or antibiotic treatments, nor probiotic supplementation for at least one month prior to enrollment and throughout the study period.

### 4.3. Vitamin D Correction and Probiotic Supplementation

Vitamin D correction was prescribed with 2000 IU/day of Vita D3 (2 tablets/day, Erba Vita Group S.p.A., San Marino, Italy) for 16 weeks only to participants with deficient (10–20 ng/mL) serum 25(OH)D concentrations at baseline, in accordance with routine clinical practice and AIFA recommendations.

Based on previous in vitro analyses [41], patients received a lyophilized probiotic mixture containing *Limosilactobacillus reuteri* LRE11 (DSM 33827), *Lacticasebacillus rhamnosus* LR04 (DSM 16605), and *Lacticasebacillus casei* LC04 (DSM 33400), at 1 × 10^9^ Active Fluorescent Units (AFU) and Colony Forming Units (CFU)/g/day with maltodextrin (Probiotical Research S.r.l., Novara, Italy) for 16 weeks. The probiotic mixture was dissolved in lukewarm water, used as an oral rinse for 30 s, and then swallowed. A telephonic follow-up was conducted every 4 weeks to ensure proper adherence to the regimen.

### 4.4. Data Collection

For each enrolled patient, a comprehensive set of variables was collected before (PRE) and after the 16-week intervention (POST). These included (i) demographic information (e.g., age, sex, body mass index (BMI)); (ii) OLP-related clinical features and histopathology (e.g., lesion form, lesion number, lesion size, affected sites); (iii) medical history, comorbidities, and ongoing medications.

Biological samples were collected at PRE and POST. At each time point, multiple specimen types were collected for each patient to enable comprehensive multi-omics analyses, including serum (SE), saliva (S, unstimulated and oral swab), OLP-lesional mucosa (L, oral swabs and photographs), non-lesional mucosa (NL, oral swabs), and feces (F).

For oral cavity-related biological samples, patients were instructed to avoid eating (including chewing gum), drinking, smoking, or performing oral hygiene for at least 2 h before each visit to prevent inaccurate results.

#### 4.4.1. Serum Samples

Two serum 2.5 mL samples were collected from each patient and time point. One sample was used for vitamin D quantification in the same hospital. The other sample was centrifuged at 3000× *g* for 10 min at room temperature (RT), aliquoted, and stored at −80 °C for cytokine and metabolomics analysis.

#### 4.4.2. Saliva Samples

For saliva collection, two mL unstimulated saliva (i.e., saliva naturally secreted by the salivary glands in the absence of any stimulation) samples were collected in the morning without oral cleaning and under fasting conditions, placed into sterile polypropylene tubes (Biosigma, Cona, Italia), kept at 4 °C, and processed within 2 h. Briefly, the saliva samples were centrifuged at 2500× *g* for 15 min at 4 °C, then aliquoted and stored at −80 °C for cytokine and proteomics analysis.

#### 4.4.3. Oral Swabs

e-NAT^®^ swabs (Copan Italia, Brescia, Italy) containing 1 mL of nucleic acid stabilizing solution were used to collect patients’ oral swabs from S, L, and NL. Samples were kept at RT and processed within 2 h by centrifugation at 1000× *g* for 2 min at RT, then stored at −80 °C until microbiome analysis.

#### 4.4.4. Stool Samples

Fecal samples were collected in appropriate containers, kept at 4 °C, and processed within 2 h. Each sample was aliquoted into 2 mL Eppendorf tubes and stored at −80 °C until gut microbiota and proteomics analysis.

### 4.5. OLP Severity and Clinical Activity

#### 4.5.1. Baseline Severity Classification

At baseline, patients were grouped into three severity categories according to lesion type, reflecting differences in inflammation and symptom burden: Severe (erosive/ulcerative lesions), Mild-Severe (papular, plaque-like, or atrophic lesions without erosive lesions), and Mild (reticular lesions only).

#### 4.5.2. Clinical Activity Assessment

For each patient, lesions were evaluated in terms of number, size, and bleeding status. These parameters were used to assess clinical activity changes during the intervention period. A composite Clinical Activity Index was constructed by mapping each marker to a numerical score (−1 for remission, 0 for stability, +1 for recurrence) and summing scores across markers for each patient. Patients were then classified as experiencing overall remission (index < 0), stability (index = 0), or recurrence (index > 0).

### 4.6. Salivary and Serum Cytokines

#### Cytokine Profiling

To assess the patient’s inflammatory status, a multiplex immunoassay was performed. Cytokine levels, including IL-1β, IL-4, IL-6, IL-10, IL-17A, IL-17F, IL-21, IL-22, IL-23, IL-25, IL-31, IL-33, IFN-γ, soluble CD40 ligand (sCD40L), tumor necrosis factor alpha (TNFα), and TGF-β1, TGF-β2, and TGF-β3, were quantified in unstimulated saliva and serum samples. Analyses were performed by the Protein Technologies Core Facility at UPO-CAAD using the Bio-Plex Pro™ Human Th17 Cytokine Panel 15-Plex and Bio-Plex™ TGF-β Assays (Bio-Rad, Segrate, Milan, Italy) and the Bio-Plex 200 System (Bio-Rad, Segrate, Milan, Italy), according to the manufacturer’s instructions.

### 4.7. Serum Metabolomics Analysis

#### 4.7.1. Sample Preparation and Nuclear Magnetic Resonance (NMR) Analysis

Serum samples were prepared and analyzed according to standard protocols [74,75]. ^1^H NMR spectra were obtained using a Bruker 600 MHz spectrometer (Bruker BioSpin, Ettlingen, Germany), operating at a proton Larmor frequency of 600.13 MHz. The spectrometer was equipped with a 5 mm PATXI ^1^H-^13^C-^15^N probe, a z-axis gradient coil, an automatic tuning and matching (ATM) unit, and an automatic refrigerated sample changer (SampleJet, Bruker BioSpin) maintained at 6 °C. Temperature stabilization during measurement was controlled by a BTO 2000 thermocouple (Bruker, Billerica, MA, USA), ensuring an accuracy of approximately 0.1 K.

Prior to data acquisition, samples were equilibrated in the NMR probe at 310 K for at least 300 s. Spectrometer calibration followed daily standard operating procedures to ensure spectral quality and reproducibility [76]. For each sample, a standard nuclear Overhauser effect spectroscopy (NOESY) sequence was applied with the following parameters: 32 scans, 98,304 data points, spectral width of 18,028 Hz, acquisition time of 2.7 s, relaxation delay of 4 s, and a mixing time of 0.01 s [77]. This setup allowed for the detection of both high molecular weight (MW) molecules (e.g., lipoproteins, proteins, lipids) and low MW metabolites. Prior to Fourier transformation, free induction decays were processed with an exponential function to achieve a line-broadening factor of 0.3 Hz. Transformed spectra were automatically corrected for phase and baseline distortions, then calibrated to the glucose anomeric doublet at δ 5.24 ppm using TopSpin 3.6.2 software (Bruker BioSpin).

#### 4.7.2. Quantification of Serum Metabolites and Lipoproteins

A total of 24 metabolites identified were quantified in all spectra using the Bruker IVDr Quantification platform for Plasma/Serum (B.I.Quant-PS™, Bruker BioSpin) [78]. Additionally, 112 lipoprotein-related parameters were analyzed with the Bruker IVDr Lipoprotein Subclass Analysis platform™ (version 1.0.0, Bruker BioSpin). This analysis covered lipoprotein components within the primary VLDL, IDL, LDL, and HDL classes, including six subclasses of VLDL (VLDL-1 to VLDL-6, ordered by increasing density and decreasing size), six LDL subclasses (LDL-1 to LDL-6), and four HDL subclasses (HDL-1 to HDL-4). For each main lipoprotein class and subclass, concentrations of triglycerides, cholesterol, free cholesterol, phospholipids, Apo-A1, Apo-A2, and Apo-B100 were calculated.

### 4.8. Saliva and Fecal Metabolomics Analysis

#### 4.8.1. Sample Preparation and MS Analysis

Analyses were performed by the Metabolism Core Facility at UPO-CAAD. To 500 μL of saliva, 700 μL of ice-cold acetonitrile was added. Samples were then centrifuged at RT at 15,000× *g* for 5 min. Supernatants were collected and transferred to a new tube, then dried in a speed vacuum and stored at −20 °C until derivatization.

Molecules were extracted from stool samples using a 1 mL mixture of acetonitrile (ACN), isopropanol (IPA), and water (3:3:2), with 5 µL of tridecanoic acid (0.5 mg/mL), 5 µL of palmitic acid d31 (0.5 mg/mL), 5 µL of stearic acid d35 (0.5 mg/mL), 3.5 µL of glycine d4 (10.07 mg/mL) as internal standards. After being vortexed, the sample was centrifuged for 15 min at 20 °C at 14,500× *g*. Next, 1 mL of the supernatant was dried in a speed vacuum at 40 °C and stored at −20 °C until derivatization.

Derivatization was performed on saliva and fecal samples with a first step of methoximation (20 µL of methoxamine at 80 °C for 20 min) and a second step of silylation (30 µL of N,O-Bis(trimethylsilyl)trifluoroacetamide at 80 °C for 20 min).

For metabolomics profiling, a LECO Pegasus BT 4D GC×GC-TOFMS system equipped with a dual-stage quad jet thermal modulator (LECO Corp., St. Joseph, MI, USA) was used. The first-dimension separation was performed on a 30 m Rxi-5Sil MS capillary column (Restek Corp., Bellefonte, PA, USA; 0.25 mm i.d., 0.25 µm film), coupled to a 2 m Rxi-17Sil MS secondary column (Restek Corp.) with identical internal diameter and film thickness. High-purity helium (99.9999%) was employed as the carrier gas at a constant flow of 1.4 mL/min. As described by Barberis et al. [79], 1 µL of each derivatized sample was injected in splitless mode at 250 °C. The primary oven temperature program was 70 °C (2 min), then ramped at 6 °C/min to 160 °C, 10 °C/min to 240 °C, and 20 °C/min to 300 °C, with a final hold of 6 min. The secondary oven was maintained at 20 °C above the primary oven, using the same temperature ramp. Electron impact ionization (70 eV) was applied, with the ion source at 250 °C. Spectra were acquired over *m*/*z* 25–550 at 200 spectra/s, with a modulation period of 4 s, an extraction frequency of 32 kHz, a modulator temperature offset of +15 °C relative to the secondary oven, and a transfer line temperature of 280 °C. Chromatograms were collected in TIC mode.

#### 4.8.2. Quantification of Salivary and Fecal Metabolites

ChromaTOF version 5.54 was used for raw data processing. Mass spectral assignment was performed by matching with NIST MS Search 2.3 libraries and the FiehnLib. In addition, an in-house library of standards was used to identify small molecules.

### 4.9. 16S rRNA Gene Sequencing

#### 4.9.1. DNA Isolation

To avoid contamination, microbial DNA extraction was performed under sterile conditions using a laminar flow cabinet and sterile reagents and materials in agreement with good scientific practices.

##### Oral Microbiota

Swabs from unstimulated saliva, OLP lesions, and non-lesional mucosa were thawed for bacterial DNA extraction using the QIAamp^®^ DNA Microbiome Kit (Qiagen, Hilden, Germany), following the manufacturer’s instructions. DNA concentration was fluorimetrically measured using the Qubit™ 1X dsDNA HS Assay Kit (Invitrogen Co., Thermo Fisher Scientific Inc., Walthman, MA, USA) on a Qubit™ 4 fluorometer (Invitrogen Co.).

##### Gut Microbiota

Stool samples were thawed at RT and subjected to bacterial DNA isolation using the QIAmp^®^ PowerFecal^®^ Pro DNA kit (Qiagen) according to the manufacturer’s instructions. Yield and quality of bacterial DNA were assessed on a NanoDrop™ One/Onec spectrophotometer (Thermo Fisher Scientifics Inc.).

#### 4.9.2. Sequencing

DNA samples (from S, L, NL, and F) were analyzed by 16S rRNA gene metabarcoding to compare microbial composition and relative abundances across the different biological matrices before (PRE) and after (POST) the intervention. Library preparation and sequencing were performed by the Integrative Genomics Core Facility at UPO-CAAD using CE-IVD kits (Arrow Diagnostics S.r.l., Genoa, Italy). The AD4SEQ Microbiota Solution A Kit was used for bacterial DNA isolated from S, L, and NL samples (V1–V3 hypervariable regions), whereas the AD4SEQ Microbiota Solution B Kit was applied to F samples (V3–V4–V6 hypervariable regions).

PCR amplification of the selected hypervariable regions was performed with degenerate primers following the manufacturer’s instructions. Amplicons were purified using Agencourt AMPure XP magnetic beads (Beckman Coulter Inc., Brea, CA, USA) and indexed in a subsequent step. DNA concentration of the libraries was quantified fluorimetrically using the Qubit™ 1X dsDNA HS Assay Kit (Invitrogen Co., Thermo Fisher Scientific Inc.) and pooled in equimolar amounts. Sequencing was carried out on a MiSeq Illumina^®^ platform with a MiSeq Reagent v2 cartridge (2 × 250 bp, paired-end) (all from Illumina, San Diego, CA, USA).

Control samples, including isolation and amplification controls, were processed in parallel with the biological samples.

#### 4.9.3. Bioinformatics Analysis

Raw sequencing data were analyzed using MicrobAT Suite v1.2.1 (SmartSeq S.r.l., Novara, Italy), a standalone software for primary analysis and quality controls. Sequences shorter than 200 nucleotides or with low quality (average Phred score < 25) were discarded [80].

High-quality sequences were aligned against the Ribosomal Database Project (RDP, RDP_trainingsetNo19_2023-08-23) to assign taxonomy, applying a query coverage ≥ 80% and a similarity threshold ≥ 97% [81]. MicrobAT generated three output files (OTU, taxonomy and metadata) as input for downstream statistical analyses.

### 4.10. Statistical Analyses

All statistical analyses were performed in R (v4.5.1).

Lesion-related outcomes (lesion number, largest lesion size in cm, and number of bleeding lesions) were evaluated in a paired POST-PRE design. Differences were assessed using two-sided Wilcoxon signed-rank tests. For descriptive reporting and visualization, each endpoint was additionally categorized per patient as remission, stability, or recurrence based on the sign of Δ = POST-PRE (remission if Δ < 0, stability if Δ = 0, and recurrence if Δ > 0). A composite Clinical Activity Index was constructed by mapping each marker to a numerical score (−1 for remission, 0 for stability, +1 for recurrence) and summing scores across markers for each patient; patients were classified as overall remission (index < 0), stability (index = 0), or recurrence (index > 0). The directional imbalance of the composite classification toward remission was evaluated using an exact binomial (sign) test comparing the number of patients in remission versus recurrence, excluding stable cases.

For cytokines (saliva and serum) and salivary and fecal metabolites, quantitative concentrations were analyzed on a log scale using log(x + offset), where the offset was defined as half of the minimum non-zero value within each analyte/feature. Serum metabolites and lipoproteins were analyzed on their original scale, as their distributions were substantially more stable compared to the other omics layers. Features detected in fewer than five samples were excluded prior to analysis.

Longitudinal changes were primarily assessed using linear mixed-effects models using lme4 (v1.1-38) [82] and, with time as the main fixed effect, adjustment for age, sex, BMI, baseline vitamin D level, baseline clinical severity, and a subject-specific random intercept to account for within-patient pairing. Model-based POST-PRE contrasts were computed with 95% confidence intervals and reported as back-transformed estimated marginal means on the original scale when appropriate (e.g., pg/mL for cytokines). For multiple testing control within each outcome family (e.g., cytokine panel, serum metabolite/lipoprotein set, salivary/fecal metabolite set, genus-level differential abundance per specimen), *p*-values were adjusted using the Benjamini–Hochberg false discovery rate (FDR) procedure, with an FDR threshold of 0.05 for declaring statistical significance.

Pathway analysis for salivary and fecal metabolomics data was performed using Metaboanalyst software v.6.0 (https://www.metaboanalyst.ca).

Microbiome analyses were conducted using MicrobAT outputs on S, L, NL, and F samples. Microbial abundances and taxonomy tables were imported into R, stored as TreeSummarizedExperiment objects, and analyzed using the mia package (v1.18) [83]. For each object separately, features classified as Eukaryota and those lacking a phylum-level taxonomic assignment were removed. To reduce sparsity and improve comparability across samples, taxa were agglomerated at the genus level. A prevalence filter was then applied to remove features present in less than 10% of samples to mitigate the impact of rare, sample-specific features that are more likely to reflect sampling stochasticity or technical artifacts than reproducible biological patterns. Relative abundances were computed and transformed using centered log-ratio (CLR) with a pseudocount equal to half of the minimum non-zero relative abundance within each dataset.

Within-sample diversity (genus richness, Shannon index, and dominance of the most abundant genus) was tested using linear mixed-effects models, including specimen type, time, and their interaction as fixed effects, a random intercept for the subject, and covariate adjustment as above. Post hoc contrasts were restricted to (i) PRE-POST comparisons within each specimen type and (ii) between-specimen comparisons within each time point, and *p*-values for these contrasts were adjusted using the Šidák procedure.

Between-sample diversity was assessed using Aitchison distances on CLR-transformed data and visualized by principal coordinate analysis (PCoA); community-level effects of specimen type and time were evaluated by PERMANOVA (adonis2 function of the vegan package v2.7-2 [84]), and homogeneity of dispersion was assessed to support the validity of PERMANOVA inferences.

Differential abundance testing was performed separately for each specimen type on genus-level CLR values using the glmmTMB package (v1.1.13) [85], with time (POST vs. PRE) as the main fixed effect, the same baseline covariates, a subject-level random intercept, and Benjamini–Hochberg FDR correction applied across genera within each specimen.

### 4.11. Multi-Omics Data Integration

Multi-omics integration was performed using Multi-Omics Factor Analysis (MOFA) [86]. The model included the following data layers (views): salivary and serum cytokines (CYT-S, CYT-Se), serum metabolites and lipoproteins (MET-Se, LIP-Se), salivary and fecal metabolomics (MET-S, MET-F), and microbiome genus-level profiles from saliva, lesional mucosa, non-lesional mucosa, and feces (16S-S, 16S-L, 16S-NL, 16S-F).

For each feature within each view, within-subject changes were computed as delta values (POST − PRE). To adjust for baseline covariates, feature-wise linear regression models were fitted with delta values as the response and age, sex, BMI, baseline vitamin D levels, and baseline disease severity as covariates; regression residuals were retained as covariate-adjusted change estimates for integration. Residuals were winsorized at the 1st and 99th percentiles within each feature to limit the influence of extreme values.

MOFA was trained on the covariate-adjusted, winsorized residual matrices using MOFA2 (v1.20.0) [86]. Model inference was initialized with a maximum of five latent factors (*num_factors* = 5), and factors explaining <5% of variance across all views were discarded during training (*drop_factor_threshold* = 0.05). Based on these settings, the final model retained three latent factors for downstream interpretation and visualization.

The fraction of variance explained was used to quantify the proportion of within-view variability captured by each latent factor. Feature-factor associations were evaluated using MOFA weights, retaining for visualization the top five features per view with |weight| > 0.1.

## 5. Conclusions

This study provides the first clinical evidence that combined probiotic supplementation and vitamin D correction can meaningfully improve the course of OLP. The integration of clinical, immunological, metabolomic and metagenomic data represents a key strength of our work, providing a multidimensional perspective on the potential mechanisms underlying the observed beneficial effects. The results suggest that this combined approach may improve OLP clinical course through a multifaceted mechanism, involving local modulation of mucosal inflammation, systemic metabolic benefits mediated by the gut-oral axis (including the production of indoles and antioxidant compounds), and favorable shifts in both oral and gut microbiota composition.

Despite their limitations, the methodology and data presented here could represent valuable support for future research and novel testable hypotheses. Larger, well-designed randomized controlled trials are warranted to confirm these observations and clarify the individual and combined effects of the interventions.

## Figures and Tables

**Table 1:** Demographic characteristics of OLP patients at enrollment (PRE).

Characteristic	N = 25 ^1^
Sex	
Female	17 (68%)
Male	8 (32%)
Age, years	68 (58, 73)
BMI, kg/m^2^	24.0 (22.5, 29.1)
Normal weight	15 (60%)
Overweight	5 (20%)
Disease severity	
Mild	9 (36%)
Mild-Severe	5 (20%)
Severe	11 (44%)

^1^ n (%); Median (first quartile, third quartile).

**Table 2:** OLP patients’ salivary cytokines. Adjusted salivary cytokine levels before (PRE) and after (POST) treatment (pg/mL). Values represent estimated marginal means (±standard error), derived from linear mixed-effects regression models controlling for baseline covariates (age, sex, BMI, vitamin D concentration, and disease severity). Fold changes (POST/PRE) are reported with 95% confidence intervals, together with the corresponding change expressed in percentage (95% CI). Although no adjusted *p*-values remained significant after multiple-testing correction, cytokines in bold displayed patterns that may justify further investigation in larger cohorts.

Cytokine (pg/mL)	TIME		Fold-Change	
	PRE ^1^	POST ^1^	POST/PRE ^2^	Change % ^2^
IFN-γ	0.28 ± 0.11	0.23 ± 0.09	0.81 (0.47, 1.43)	−19 (−53, 43)
IL-10	0.22 ± 0.08	0.25 ± 0.08	1.12 (0.56, 2.21)	12 (−44, 121)
IL-17A	0.18 ± 0.07	0.09 ± 0.04	0.53 (0.23, 1.21)	−47 (−77, 21)
IL-17F	11.26 ± 4.41	6.75 ± 2.64	0.60 (0.34, 1.06)	−40 (−66, 6)
IL-1β	134.60 ± 39.46	89.63 ± 26.28	0.67 (0.40, 1.11)	−33 (−60, 11)
**IL-21**	7.17 ± 2.76	4.19 ± 1.61	0.58 (0.35, 0.98)	−42 (−65, −2)
IL-22	15.59 ± 8.25	6.37 ± 3.37	0.41 (0.14, 1.20)	−59 (−86, 20)
IL-23	2.24 ± 1.27	1.98 ± 1.13	0.88 (0.35, 2.23)	−12 (−65, 123)
IL-25	0.68 ± 0.37	0.29 ± 0.16	0.43 (0.19, 1.00)	−57 (−81, 0)
IL-31	25.34 ± 13.82	17.98 ± 9.80	0.71 (0.26, 1.94)	−29 (−74, 94)
**IL-33**	2.54 ± 1.46	0.99 ± 0.57	0.39 (0.18, 0.82)	−61 (−82, −18)
IL-4	0.83 ± 0.35	0.45 ± 0.19	0.54 (0.27, 1.08)	−46 (−73, 8)
IL-6	11.86 ± 3.88	11.30 ± 3.70	0.95 (0.64, 1.41)	−5 (−36, 41)
sCD40L	2.95 ± 0.65	2.31 ± 0.51	0.79 (0.53, 1.16)	−21 (−47, 16)
TGF-β1	94.14 ± 33.15	83.54 ± 29.41	0.89 (0.41, 1.90)	−11 (−59, 90)
TGF-β2	47.07 ± 16.00	38.01 ± 12.92	0.81 (0.54, 1.21)	−19 (−46, 21)
TGF-β3	0.97 ± 0.27	0.58 ± 0.16	0.60 (0.31, 1.16)	−40 (−69, 16)
TNF-α	25.89 ± 8.03	19.94 ± 6.19	0.77 (0.47, 1.25)	−23 (−53, 25)

^1^ Estimated marginal mean ± standard error (pg/mL). ^2^ Average change % (95% CI).

**Table 3:** OLP patients’ serum cytokines. Adjusted serum cytokine levels before (PRE) and after (POST) treatment (pg/mL). Values represent estimated marginal means (±standard error), derived from linear mixed-effects regression models controlling for baseline covariates (age, sex, BMI, vitamin D concentration, and disease severity). Fold changes (POST/PRE) are reported with 95% confidence intervals, together with the corresponding change expressed in percentage (95% CI).

Cytokine (pg/mL)	TIME		Fold Change	
	PRE ^1^	POST ^1^	POST/PRE ^2^	Change % ^2^
IFN-γ	1.09 ± 0.35	1.19 ± 0.38	1.09 (0.95, 1.25)	9 (−5, 25)
IL-10	1.92 ± 0.50	1.66 ± 0.43	0.86 (0.69, 1.08)	−14 (−31, 8)
IL-17F	7.76 ± 1.56	7.62 ± 1.54	0.98 (0.95, 1.01)	−2 (−5, 1)
IL-1β	0.02 ± 0.01	0.02 ± 0.01	1.00 (0.97, 1.04)	0 (−3, 4)
IL-21	9.96 ± 2.03	10.50 ± 2.14	1.05 (0.95, 1.17)	5 (−5, 17)
IL-25	0.64 ± 0.17	0.63 ± 0.17	0.99 (0.96, 1.02)	−1 (−4, 2)
IL-33	6.15 ± 1.64	5.95 ± 1.58	0.97 (0.87, 1.07)	−3 (−13, 7)
IL-4	0.40 ± 0.13	0.31 ± 0.10	0.79 (0.57, 1.09)	−21 (−43, 9)
IL-6	0.24 ± 0.10	0.28 ± 0.12	1.17 (0.77, 1.76)	17 (−23, 76)
sCD40L	3.86 ± 1.00	3.07 ± 0.79	0.79 (0.56, 1.13)	−21 (−44, 13)
TGF-β1	39,806.69 ± 2828.23	38,234.19 ± 2716.51	0.96 (0.88, 1.05)	−4 (−12, 5)
TGF-β2	1161.13 ± 26.17	1116.71 ± 25.17	0.96 (0.93, 1.00)	−4 (−7, 0)
TGF-β3	469.82 ± 12.74	472.72 ± 12.82	1.01 (0.96, 1.05)	1 (−4, 5)
TNF-α	1.86 ± 0.56	1.44 ± 0.43	0.77 (0.41, 1.46)	−23 (−59, 46)

^1^ Estimated marginal mean ± standard error (pg/mL) ^2^ Average change % (95% CI).

## Data Availability

The dataset supporting the conclusions of this article is available in the “MICROCOSM in vivo study data” repository [https://doi.org/10.5281/zenodo.19188548].

## Supplementary Materials

The following supporting information can be downloaded at: https://www.mdpi.com/article/10.3390/ijms27156707/s1.

## Abbreviations

The following abbreviations are used in this manuscript:

OLP	oral lichen planus
NF-κB	nuclear factor-kappa B
MMPs	Matrix metalloproteinases
WHO	World Health Organization
OSCC	oral squamous cell carcinoma
Treg	regulatory T cells
Th17	T-helper 17
IL	interleukin
TGF-β	Transforming Growth Factor-beta
CFSs	Cell Free Supernatants
Th1	T-helper 1
IFN-γ	interferon-gamma
CEI	Comitato Etico Interaziendale
AOU	Azienda Ospedaliero-Universitaria
IRB	Institutional Review Board
LRE11	Limosilactobacillus reuteri
LR04	Lacticasebacillus rhamnosus
LC04	Lacticasebacillus casei
AFU	Active Fluorescent Units
CFU	Colony Forming Units
s	seconds
BMI	body mass index
SE	serum
S	saliva
L	OLP-lesioned mucosa
NL	non-lesioned healthy mucosa
F	feces
min	minutes
RT	room temperature
h	hours
sCD40L	soluble CD40 ligand
TNFα	tumor necrosis factor alpha
NMR	nuclear magnetic resonance
ATM	automatic tuning and matching
NOESY	nuclear Overhauser effect spectroscopy
MW	molecular weight
ACN	acetonitrile
IPA	isopropanol
RDP	Ribosomal Database Project
FDR	false discovery rate
CLR	centered log-ratio
PCoA	principal component analysis
MOFA	Multi-Omics Factor Analysis
CYT-S	salivary cytokines
CYT-Se	serum cytokines
MET-Se	serum metabolites
LIP-Se	serum lipoproteins
MET-S	salivary metabolomics
MET-F	fecal metabolomics
16S-S	salivary microbiome
16S-L	lesional microbiome
16S-NL	non-lesional microbiome
16S-F	fecal microbiome
AIFA	Agenzia Italiana del Farmaco
