# Supplementary material for: Effects of Probiotics and Vitamin D Deficiency Correction on Clinical, Inflammatory, Metabolic, and Microbiota Profiles in Older Adults with Oral Lichen Planus: A Multi-Omics Study
Source: Int J Mol Sci. 2026 Jul 27;27(15):6707. doi: 10.3390/ijms27156707 (PMC13467221; doi:10.3390/ijms27156707)
Supplement: Supplementary file 1 [file ijms-27-06707-s001.zip › ijms-4357436-supplementary.pdf]

Effects of Probiotics and Vitamin D Deficiency Correction on Clinical, Inflammatory, Metabolic, and Microbiota Profiles in Older Adults with Oral Lichen Planus: A Multi-Omics Study

Supplementary material

Content

This file provides supplementary figures and tables supporting the results reported in the main manuscript. Data are organized into five thematic sections reflecting the multi-omics design of the MICROCOSM study: clinical characterization of the OLP cohort (Tables S1-S6), systemic metabolic and lipoprotein assessment (Tables S7-S8), saliva and feces metabolomic signatures (Tables S9-S10), and oral and gut microbiome characterization (Figure S1; Tables S11-S15).

For further details on study design, sample processing, and statistical analyses, please refer to the Materials and Methods section of the main manuscript.

Clinical informations

Table S1: Distribution of oral lesions by localization and type across overall cohort and clinical severity groups. Lesion types include reticular, papular/plaque-like, atrophic, erosive, and combinations thereof. Severity groups are classified as Mild, Mild–Severe, and Severe.

**Localization and type**	**Overall** N = 25[1]	**Mild** N = 9[1]	**Mild-Severe** N = 5[1]	**Severe** N = 11[1]
**Cheeks**				
No	0 (0%)	0 (0%)	0 (0%)	0 (0%)
Reticular	20 (80%)	9 (100%)	3 (60%)	8 (73%)
Papular + Plaque like	3 (12%)	0 (0%)	2 (40%)	1 (9%)
Erosive	2 (4.0%)	0 (0%)	0 (0%)	2 (18%)
**Tongue**				
No	17 (68%)	8 (89%)	1 (20%)	8 (73%)
Reticular	2 (8.0%)	1 (11%)	0 (0%)	1 (9%)
Papular + Plaque like	2 (8.0%)	0 (0%)	2 (40%)	0 (0%)
Atrophic	3 (12%)	0 (0%)	2 (40%)	1 (9%)
Erosive	1 (4.0%)	0 (0%)	0 (0%)	1 (9%)
**Gingiva**				
No	12 (48%)	9 (100%)	3 (60%)	0 (0%)
Papular + Plaque like	2 (8.0%)	0 (0%)	2 (40%)	0 (0%)
Erosive	11 (44%)	0 (0%)	0 (0%)	11 (100%)

[1] n (%)

Table S2: Distribution of potentially OLP-related patients' comorbidities.

**Comorbities**	**N = 25**[1]
Hypertension	15 (60%)
Diabetes	7 (28%)
Hypothyroidism	4 (16%)
Psychosis	4 (16%)
Hepatitis C Virus	1 (4%)
None	5 (20%)

[1] n (%)

Table S3: Distribution of patients' comorbidities (not OLP-related).

**Comorbidities**	**N = 25**[1]
Osteoporosis	8 (32%)
Blood diseases	5 (20%)
Cardiovascular diseases	5 (20%)
Solid tumor	5 (20%)
Dermatomucosites	4 (16%)
Dyslipidemia	4 (16%)
Respiratory system diseases	3 (12%)
Genetic diseases	2 (8%)
Hepatitis B Virus	2 (8%)
Acromegaly	1 (4%)
Autoimmune connective tissue disease	1 (4%)
Fibromyalgia	1 (4%)
Hypovitaminosis D	1 (4%)
Neurological diseases	1 (4%)
None	7 (28%)

[1] n (%)

Table S4: Distribution of systemic medications potentially associated with OLP

Drug type	N = 25[1]
Anti-hypertensives	15 (60%)
Oral hypoglycemics	5 (20%)
None	10 (40%)

[1] n (%)

Table S5: Distribution of systemic medications related to a decrease in the salivary flow.

Drug type	N = 25[1]
Anti-hypertensives	15 (60%)
Antacids	5 (20%)
Psychiatric drugs	4 (16%)
Diuretic	3 (12%)
Bronchodilators	2 (8%)
None	7 (28%)

[1] n (%)

Table S6: Vitamin D supplementation types and serum concentrations at the baseline (PRE). Data are presented according to supplementation status: chronic users, supplementation initiated during the study, and no supplementation. Serum 25(OH)D concentrations (ng/mL) are reported as median (first quartile, third quartile), with classification into deficient (10-20 ng/mL), normal (20-40 ng/mL), and high (40-100 ng/mL) categories.

	**Chronic**<br>N = 8[1]	**From the study**<br>N = 7[1]	**No**<br>N = 10[1]
**Type**			
Dibase (various posologies)	7 (88%)	-	-
Didrogyl (400 U/week)	1 (13%)	-	-
Erba Vita (2000 U/die)	-	7 (100%)	-
**Concentration**, ng/mL	41 (30, 47)	15 (11, 21)	30 (28, 35)
Deficient (<20 ng/mL)	0 (0%)	5 (71%)	0 (0%)
Normal (20-40 ng/mL)	4 (50%)	2 (29%)	9 (90%)
High (40-100 ng/mL)	4 (50%)	0 (0%)	1 (10%)

[1] n (%); Median (first quartile, third quartile)

Serum metabolites and lipoproteins

Table S7. OLP patients' serum metabolites. Adjusted serum metabolite quantifications before (PRE) and after (POST) treatment. Values represent estimated marginal means (± standard error), derived from linear mixed-effects regression models controlling for baseline covariates (age, sex, BMI, Vitamin D concentration, and disease severity). Differences (POST-PRE) are reported with 95% confidence intervals. Although no adjusted p-values remained significant after multiple-testing correction, metabolite in bold displayed a pattern that may warrant further investigation in larger cohorts.

	**TIME**		**DIFFERENCE**
	**PRE**[1]	**POST**[1]	**POST - PRE**[2]
Acetate	0.04 ± 0.01	0.04 ± 0.01	-0.01 (-0.02, 0.01)
Acetone	0.03 ± 0.00	0.02 ± 0.00	-0.01 (-0.02, 0.00)
**Alanine**	**0.46 ± 0.02**	**0.50 ± 0.02**	**0.05 (0.00, 0.09)**
Citrate	0.19 ± 0.01	0.18 ± 0.01	-0.01 (-0.03, 0.00)
Creatine	0.01 ± 0.00	0.01 ± 0.00	0.00 (-0.00, 0.01)
Creatinine	0.09 ± 0.01	0.09 ± 0.01	-0.00 (-0.02, 0.01)
Dimethylsulfone	0.01 ± 0.00	0.01 ± 0.00	0.00 (-0.00, 0.00)
Formate	0.02 ± 0.00	0.02 ± 0.00	-0.00 (-0.00, 0.00)
Glucose	8.05 ± 0.63	7.13 ± 0.63	-0.93 (-2.08, 0.23)
Glutamine	0.73 ± 0.02	0.76 ± 0.02	0.03 (-0.00, 0.06)
GlycA	6358.10 ± 225.78	6482.85 ± 225.78	124.75 (-127.90, 377.40)
GlycB	1260.95 ± 60.17	1257.89 ± 60.17	-3.06 (-59.54, 53.42)
Glycine	0.25 ± 0.01	0.26 ± 0.01	0.01 (-0.01, 0.03)
Histidine	0.07 ± 0.00	0.08 ± 0.00	0.00 (-0.00, 0.01)
Isoleucine	0.06 ± 0.00	0.07 ± 0.00	0.00 (-0.01, 0.01)
Lactate	2.52 ± 0.11	2.57 ± 0.11	0.04 (-0.19, 0.27)
Leucine	0.11 ± 0.01	0.12 ± 0.01	0.00 (-0.01, 0.01)
N,N-Dimethylglycine	0.01 ± 0.00	0.01 ± 0.00	0.00 (-0.00, 0.00)
Phenylalanine	0.05 ± 0.00	0.05 ± 0.00	-0.00 (-0.01, 0.00)
Pyruvate	0.08 ± 0.01	0.08 ± 0.01	-0.00 (-0.02, 0.02)
Succinate	0.01 ± 0.00	0.01 ± 0.00	-0.00 (-0.00, 0.00)
Trimethylamine-N-oxide	0.04 ± 0.00	0.03 ± 0.00	-0.01 (-0.02, 0.00)
Tyrosine	0.06 ± 0.00	0.07 ± 0.00	0.00 (-0.00, 0.01)
Valine	0.27 ± 0.01	0.28 ± 0.01	0.01 (-0.01, 0.03)

[1] Estimated average ratio ± standard error
[2] Average difference (95% CI)

Table S8. OLP patients' serum lipoproteins. Adjusted lipoprotein quantifications before (PRE) and after (POST) treatment. Lipoproteins are organized by main fractions (apolipoproteins, VLDL, IDL, LDL, HDL) and their respective subfractions. Values represent estimated marginal means (± standard error), derived from linear mixed-effects regression models controlling for baseline covariates (age, sex, BMI, Vitamin D concentration, and disease severity). Differences (POST-PRE) are reported with 95% confidence intervals. Although no adjusted p-values remained significant after multiple-testing correction, lipoproteins in bold displayed patterns that may warrant further investigation in larger cohorts.

	**TIME**		**DIFFERENCE**
	**PRE**[1]	**POST**[1]	**POST - PRE**[2]
**Apolipoproteins**			
Apo A1	150.88 ± 4.39	154.60 ± 4.39	3.72 (-4.48, 11.93)
Apo A1 HDL	153.42 ± 4.62	157.57 ± 4.62	4.14 (-4.36, 12.65)
Apo A1 HDL1	27.76 ± 3.00	31.53 ± 3.00	3.77 (-0.08, 7.63)
Apo A1 HDL2	20.22 ± 0.82	20.76 ± 0.82	0.54 (-0.99, 2.07)
Apo A1 HDL3	27.47 ± 1.09	27.71 ± 1.09	0.24 (-1.64, 2.12)
Apo A1 HDL4	78.98 ± 2.97	78.16 ± 2.97	-0.82 (-4.29, 2.65)
Apo A2	32.62 ± 1.08	32.51 ± 1.08	-0.11 (-1.82, 1.60)
Apo A2 HDL	33.20 ± 1.04	33.07 ± 1.04	-0.13 (-1.82, 1.55)
Apo A2 HDL1	2.70 ± 0.26	3.06 ± 0.26	0.37 (-0.07, 0.81)
Apo A2 HDL2	3.44 ± 0.16	3.62 ± 0.16	0.18 (-0.18, 0.54)
Apo A2 HDL3	6.21 ± 0.27	6.24 ± 0.27	0.03 (-0.46, 0.53)
Apo A2 HDL4	20.12 ± 0.94	19.48 ± 0.94	-0.64 (-1.58, 0.31)
Apo B100	78.57 ± 4.97	76.56 ± 4.97	-2.01 (-8.65, 4.62)
Apo B100 / Apo A1	0.52 ± 0.03	0.50 ± 0.03	-0.02 (-0.06, 0.02)
ApoB Total Particle Number	1428.66 ± 90.40	1392.01 ± 90.40	-36.65 (-157.24, 83.94)
Apo B LDL	64.92 ± 4.45	62.90 ± 4.45	-2.02 (-8.27, 4.24)
Apo B LDL1	10.88 ± 0.63	9.88 ± 0.63	-1.00 (-2.05, 0.06)
Apo B LDL2	7.94 ± 0.73	7.60 ± 0.73	-0.33 (-1.44, 0.77)
Apo B LDL3	6.55 ± 0.72	6.21 ± 0.72	-0.35 (-1.39, 0.70)
Apo B LDL4	7.72 ± 1.05	7.57 ± 1.05	-0.15 (-1.56, 1.26)
Apo B LDL5	11.79 ± 1.41	12.02 ± 1.41	0.23 (-1.39, 1.86)
Apo B LDL6	20.25 ± 1.55	19.93 ± 1.55	-0.32 (-2.35, 1.71)
Apo B IDL	4.47 ± 0.43	4.21 ± 0.43	-0.25 (-0.92, 0.42)
Apo B VLDL	7.42 ± 0.78	8.18 ± 0.78	0.76 (-0.54, 2.05)
**Cholesterol**			
Cholesterol HDL	59.31 ± 2.71	61.04 ± 2.71	1.73 (-2.84, 6.31)
Cholesterol HDL1	16.92 ± 1.76	18.70 ± 1.76	1.78 (-0.65, 4.20)
Cholesterol HDL2	8.43 ± 0.56	8.80 ± 0.56	0.37 (-0.44, 1.18)
Cholesterol HDL3	10.43 ± 0.52	10.36 ± 0.52	-0.07 (-0.86, 0.72)
Cholesterol HDL4	21.41 ± 0.96	20.93 ± 0.96	-0.48 (-1.68, 0.73)
Cholesterol LDL	95.92 ± 6.90	94.41 ± 6.90	-1.51 (-10.89, 7.88)
Cholesterol LDL1	19.43 ± 1.22	17.96 ± 1.22	-1.47 (-3.62, 0.67)

	TIME		DIFFERENCE
	**PRE**[1]	**POST**[1]	**POST - PRE**[2]
Cholesterol LDL2	13.58 ± 1.49	13.29 ± 1.49	-0.29 (-2.58, 1.99)
Cholesterol LDL3	10.53 ± 1.31	10.25 ± 1.31	-0.28 (-2.26, 1.71)
Cholesterol LDL4	11.65 ± 1.69	11.56 ± 1.69	-0.09 (-2.39, 2.21)
Cholesterol LDL5	16.40 ± 2.08	17.05 ± 2.08	0.65 (-1.69, 3.00)
Cholesterol LDL6	24.04 ± 1.97	24.11 ± 1.97	0.07 (-2.35, 2.49)
Cholesterol IDL	8.80 ± 1.27	8.72 ± 1.27	-0.08 (-2.10, 1.94)
Cholesterol VLDL	16.56 ± 2.12	19.37 ± 2.12	2.81 (-0.99, 6.60)
Cholesterol VLDL1	5.31 ± 0.64	6.39 ± 0.64	1.08 (-0.19, 2.36)
Cholesterol VLDL2	2.37 ± 0.34	2.82 ± 0.34	0.45 (-0.21, 1.10)
Cholesterol VLDL3	2.53 ± 0.49	2.87 ± 0.49	0.35 (-0.53, 1.22)
Cholesterol VLDL4	4.52 ± 0.72	4.79 ± 0.72	0.27 (-0.78, 1.32)
Cholesterol VLDL5	1.70 ± 0.16	1.86 ± 0.16	0.16 (-0.10, 0.42)
**Free Cholesterol**			
Free Cholesterol HDL	12.56 ± 0.72	13.19 ± 0.72	0.62 (-0.56, 1.81)
Free Cholesterol HDL1	4.05 ± 0.42	4.41 ± 0.42	0.36 (-0.27, 0.99)
Free Cholesterol HDL2	1.87 ± 0.13	1.91 ± 0.13	0.03 (-0.16, 0.23)
Free Cholesterol HDL3	2.01 ± 0.13	2.02 ± 0.13	0.01 (-0.22, 0.23)
Free Cholesterol HDL4	3.75 ± 0.26	3.55 ± 0.26	-0.19 (-0.46, 0.07)
Free Cholesterol LDL	27.92 ± 1.87	27.20 ± 1.87	-0.72 (-3.25, 1.82)
Free Cholesterol LDL1	5.94 ± 0.40	5.53 ± 0.40	-0.41 (-1.03, 0.21)
Free Cholesterol LDL2	4.49 ± 0.49	4.33 ± 0.49	-0.16 (-0.88, 0.57)
Free Cholesterol LDL3	3.90 ± 0.42	3.65 ± 0.42	-0.25 (-0.89, 0.38)
Free Cholesterol LDL4	3.71 ± 0.40	3.60 ± 0.40	-0.12 (-0.70, 0.46)
Free Cholesterol LDL5	4.64 ± 0.51	4.59 ± 0.51	-0.06 (-0.63, 0.52)
Free Cholesterol LDL6	5.90 ± 0.45	5.79 ± 0.45	-0.11 (-0.63, 0.40)
Free Cholesterol IDL	2.62 ± 0.36	2.55 ± 0.36	-0.07 (-0.64, 0.51)
Free Cholesterol VLDL	8.06 ± 0.91	9.32 ± 0.91	1.26 (-0.31, 2.83)
Free Cholesterol VLDL1	2.00 ± 0.32	2.50 ± 0.32	0.50 (-0.01, 1.01)
Free Cholesterol VLDL2	0.88 ± 0.15	1.10 ± 0.15	0.22 (-0.07, 0.51)
Free Cholesterol VLDL3	1.08 ± 0.20	1.28 ± 0.20	0.20 (-0.17, 0.58)
Free Cholesterol VLDL4	1.67 ± 0.31	1.83 ± 0.31	0.16 (-0.35, 0.67)
Free Cholesterol VLDL5	0.79 ± 0.09	0.85 ± 0.09	0.06 (-0.13, 0.26)
**Phospolipids**			
Phospholipids HDL	82.80 ± 3.28	84.95 ± 3.28	2.16 (-3.43, 7.75)
Phospholipids HDL1	21.29 ± 2.08	23.59 ± 2.08	2.29 (-0.43, 5.01)
Phospholipids HDL2	13.41 ± 0.73	14.02 ± 0.73	0.61 (-0.56, 1.78)
Phospholipids HDL3	17.23 ± 0.77	17.26 ± 0.77	0.03 (-1.25, 1.31)
Phospholipids HDL4	29.91 ± 1.24	29.41 ± 1.24	-0.50 (-1.94, 0.95)
Phospholipids LDL	56.26 ± 3.48	54.92 ± 3.48	-1.34 (-6.12, 3.43)
Phospholipids LDL1	11.67 ± 0.64	10.76 ± 0.64	-0.91 (-1.99, 0.18)
Phospholipids LDL2	7.90 ± 0.77	7.66 ± 0.77	-0.24 (-1.42, 0.93)

	TIME		DIFFERENCE
	**PRE**[1]	**POST**[1]	**POST - PRE**[2]
Phospholipids LDL3	6.41 ± 0.69	6.10 ± 0.69	-0.31 (-1.33, 0.72)
Phospholipids LDL4	6.76 ± 0.86	6.68 ± 0.86	-0.07 (-1.25, 1.10)
Phospholipids LDL5	9.10 ± 1.04	9.41 ± 1.04	0.32 (-0.84, 1.48)
Phospholipids LDL6	13.63 ± 0.98	13.69 ± 0.98	0.06 (-1.14, 1.27)
Phospholipids IDL	5.58 ± 0.60	6.20 ± 0.60	0.62 (-0.45, 1.70)
Phospholipids VLDL	17.67 ± 2.13	20.88 ± 2.13	3.21 (-0.39, 6.81)
Phospholipids VLDL1	**4.56 ± 0.66**	**5.64 ± 0.66**	**1.08 (0.02, 2.15)**
Phospholipids VLDL2	2.48 ± 0.36	3.01 ± 0.36	0.53 (-0.09, 1.16)
Phospholipids VLDL3	2.86 ± 0.47	3.40 ± 0.47	0.54 (-0.26, 1.33)
Phospholipids VLDL4	4.27 ± 0.55	4.64 ± 0.55	0.36 (-0.49, 1.21)
Phospholipids VLDL5	1.89 ± 0.19	2.16 ± 0.19	0.28 (-0.04, 0.59)
**Triglycerides (TG)**			
TG	104.21 ± 8.17	117.06 ± 8.17	12.85 (-2.38, 28.09)
Total Cholesterol	181.75 ± 8.97	184.14 ± 8.97	2.38 (-11.89, 16.65)
Triglycerides HDL	9.98 ± 0.72	10.52 ± 0.72	0.54 (-0.55, 1.64)
Triglycerides HDL1	3.16 ± 0.33	3.45 ± 0.33	0.29 (-0.12, 0.69)
Triglycerides HDL2	1.59 ± 0.13	1.71 ± 0.13	0.12 (-0.09, 0.34)
Triglycerides HDL3	1.99 ± 0.15	2.05 ± 0.15	0.06 (-0.19, 0.31)
Triglycerides HDL4	3.42 ± 0.27	3.44 ± 0.27	0.02 (-0.33, 0.36)
**Triglycerides LDL**	**18.42 ± 0.97**	**16.71 ± 0.97**	**-1.71 (-3.13, -0.29)**
Triglycerides LDL1	**5.72 ± 0.37**	**4.88 ± 0.37**	**-0.84 (-1.35, -0.33)**
Triglycerides LDL2	1.98 ± 0.13	1.77 ± 0.13	-0.22 (-0.45, 0.02)
Triglycerides LDL3	2.54 ± 0.15	2.42 ± 0.15	-0.12 (-0.35, 0.11)
Triglycerides LDL4	2.16 ± 0.20	1.94 ± 0.20	-0.22 (-0.49, 0.05)
Triglycerides LDL5	2.63 ± 0.26	2.58 ± 0.26	-0.06 (-0.42, 0.30)
Triglycerides LDL6	4.27 ± 0.28	4.22 ± 0.28	-0.05 (-0.44, 0.34)
**Triglycerides IDL**	**8.83 ± 1.29**	**11.22 ± 1.29**	**2.39 (0.04, 4.74)**
Triglycerides VLDL	62.77 ± 7.24	74.33 ± 7.24	11.57 (-0.98, 24.12)
Triglycerides VLDL1	**28.15 ± 4.08**	**34.97 ± 4.08**	**6.82 (0.33, 13.31)**
Triglycerides VLDL2	8.85 ± 1.45	11.23 ± 1.45	2.38 (-0.10, 4.86)
Triglycerides VLDL3	7.81 ± 1.36	9.48 ± 1.36	1.67 (-0.67, 4.01)
Triglycerides VLDL4	8.01 ± 1.11	8.90 ± 1.11	0.88 (-0.80, 2.57)
Triglycerides VLDL5	3.08 ± 0.20	3.34 ± 0.20	0.26 (-0.05, 0.58)
LDL Particle Number	1180.43 ± 80.84	1143.74 ± 80.84	-36.69 (-150.44, 77.07)
LDL1 Particle Number	197.71 ± 11.50	179.61 ± 11.50	-18.10 (-37.29, 1.08)
LDL2 Particle Number	144.36 ± 13.34	138.28 ± 13.34	-6.09 (-26.15, 13.98)
LDL3 Particle Number	119.10 ± 13.13	112.82 ± 13.13	-6.28 (-25.33, 12.78)
LDL4 Particle Number	140.28 ± 19.14	137.60 ± 19.14	-2.68 (-28.40, 23.04)
LDL5 Particle Number	214.34 ± 25.72	218.57 ± 25.72	4.23 (-25.30, 33.75)
LDL6 Particle Number	368.27 ± 28.25	362.45 ± 28.25	-5.82 (-42.67, 31.03)

	TIME		DIFFERENCE
	**PRE**[1]	**POST**[1]	**POST - PRE**[2]
IDL Particle Number	81.19 ± 7.85	76.59 ± 7.85	-4.60 (-16.79, 7.59)
VLDL Particle Number	134.93 ± 14.15	148.66 ± 14.15	13.73 (-9.78, 37.24)
LDL / HDL	1.59 ± 0.11	1.54 ± 0.11	-0.05 (-0.18, 0.09)

[1] Estimated average ratio ± standard error

[2] Average difference (95% CI)

Saliva and feces metabolites

Table S9. OLP patients' salivary metabolites. Differentially abundant salivary metabolites between baseline (PRE) and after (POST) treatment quantifications ($p<0.05$). Values represent fold changes (POST/PRE ratio) in logarithmic scale, p-values (adjusted for multiple testing), and directions, derived from linear mixed-effects regression models controlling for baseline covariates (age, sex, BMI, Vitamin D concentration, and disease severity).

Metabolite	logFC	P-value	Direction
Propanedioic acid	6.18947	2.74E-05	POST > PRE
Myristic acid	4.07151	0.001882	POST > PRE
4-Pyridinol	3.60688	0.00412	POST > PRE
Diglycerol	3.58774	0.000357	POST > PRE
Carbamic acid	2.97812	0.009483	POST > PRE
Benzene, (1-methyldodecyl)-	2.83516	0.000252	POST > PRE
Xylitol	2.79095	0.012804	POST > PRE
2-Isopropyl-5-methyl-1-heptanol	2.74818	0.003438	POST > PRE
2-Ethylhexanol	2.63382	0.046545	POST > PRE
Hydroxyethylthiazole	2.6167	0.00822	POST > PRE
3-tert-Butyl-4-penten-2-one	2.29812	0.042275	POST > PRE
L-Aspartic acid	2.27316	0.0175	POST > PRE
Octadecadienoic acid	2.22684	0.002439	POST > PRE
Heptadecanoic acid	2.09475	0.027378	POST > PRE
3-Deoxyhexitol	1.72613	0.005899	POST > PRE
Hippuric acid	1.57171	0.046545	POST > PRE
Theobromine	-0.949347	0.047206	POST < PRE
1,3-Diacetin	-0.995947	0.047383	POST < PRE
Cyclo(Pro-Val)	-1.090786	0.049573	POST < PRE
Methyl hexadecanoic acid	-1.277735	0.007983	POST < PRE
2-Methylglutaconic acid	-1.327108	0.046579	POST < PRE
1,2-Dimethoxy-4-(1-methoxyethenyl)benzene	-1.327423	0.024555	POST < PRE
Erythronolactone	-1.357036	0.042275	POST < PRE
1,5-Anhydroglucitol	-1.376238	0.025338	POST < PRE
1,2,3-Butanetriol	-1.382393	0.023024	POST < PRE
Ribitol	-1.429657	0.008541	POST < PRE
1-Dodecanol	-1.440652	0.024208	POST < PRE
3-Pyridinol	-1.463352	0.005899	POST < PRE
Acetamide, N-ethyl-	-1.47247	0.01535	POST < PRE
Isopropyl myristate	-1.496506	0.013481	POST < PRE

Metabolite	logFC	P-value	Direction
Diacetin	-1.520747	0.022914	POST < PRE
2,4-Dimethylacetophenone	-1.525357	0.012804	POST < PRE
2,5-Dimethoxyacetophenone	-1.547204	0.021971	POST < PRE
N,N-Dimethylglycine	-1.570556	0.013481	POST < PRE
2,6-Di-tert-butyl-4-hydroxy-4-methylcyclohexa-2,5-dien-1-one	-1.581121	0.047206	POST < PRE
(2,5-Dimethoxyphenyl)acetone	-1.581471	0.027378	POST < PRE
1,1'-Biphenyl, 3,4-diethyl-	-1.604698	0.027378	POST < PRE
2',5'-Dimethoxypropiophenone	-1.626552	0.00412	POST < PRE
2,2,4-Trimethyl-1,3-pentanediol diisobutyrate	-1.637294	0.001332	POST < PRE
Cholest-5-en-3-ol (3beta)-, carbonochloridate	-1.651794	0.020406	POST < PRE
Glycolic acid	-1.654551	0.036039	POST < PRE
Ethyl stearate	-1.662536	0.012804	POST < PRE
7-Methoxy-2,2,4-trimethyl-1,2-dihydroquinoline	-1.664031	0.018043	POST < PRE
L-Valine	-1.671739	0.036039	POST < PRE
Isopropyl palmitate	-1.707474	0.021997	POST < PRE
Tetrahydrogeranyl formate	-1.772381	0.00412	POST < PRE
D-Fucitol	-1.772998	0.036233	POST < PRE
Terephthalic acid	-1.774527	0.013287	POST < PRE
Anthranilic acid	-1.774854	0.047206	POST < PRE
Palmitic Acid	-1.815016	0.011941	POST < PRE
p-Hydroxydiisopropylbenzene	-1.825646	0.000236	POST < PRE
13-Docosenamide	-1.851926	0.020406	POST < PRE
β-D-Glucopyranose, 2-(acetylamino)-3,6-anhydro-2-deoxy	-1.939566	0.02227	POST < PRE
1-Decanol, 2-hexyl-	-1.948822	6.01E-06	POST < PRE
L-Leucine	-1.989591	0.011941	POST < PRE
Benzene, 1,4-dimethoxy-	-2.04249	0.00412	POST < PRE
2,5-Dimethoxybenzoic acid	-2.06561	0.013481	POST < PRE
2H-Pyran-2-one, tetrahydro-	-2.095352	0.02987	POST < PRE
D-Mannose	-2.125784	0.001642	POST < PRE
Theophylline	-2.129156	0.00412	POST < PRE
Eicosanoic acid	-2.134571	0.042275	POST < PRE
1,3-Dioxolane, 2-heptyl-	-2.15425	0.030379	POST < PRE
N-(3-(2-Oxopyrrolidin-1-yl)propyl)acetamide	-2.241918	0.00412	POST < PRE
Glyoxime	-2.268644	0.035131	POST < PRE
Benzoic Acid	-2.306157	0.000357	POST < PRE

**Metabolite**	**logFC**	**P-value**	**Direction**
Octadecanamide	-2.318841	0.027378	POST < PRE
2(3H)-Furanone, dihydro-5-tetradecyl-	-2.473628	0.000357	POST < PRE
2-Hydroxybutyric acid	-2.476389	0.039598	POST < PRE
2H-Pyran-2-one, tetrahydro-6-undecyl-	-2.518224	0.003438	POST < PRE
1H-Indole, 5-methoxy-2-methyl-	-2.560846	0.02651	POST < PRE
Acetamide, N-(2,6-dioxo-3-beta-piperidyl)-2-phenyl-, sodium salt	-2.613571	0.012804	POST < PRE
2,4-Thiazolidinedione	-2.663349	0.002994	POST < PRE
Hexadecanamide	-2.738464	0.002439	POST < PRE
D-Arabinose	-2.79638	0.00412	POST < PRE
beta-D-(+)-Xylopyranose	-2.93217	0.007262	POST < PRE
1-Deoxypentitol	-2.974964	0.012804	POST < PRE
4-Hydroxyacetophenone	-3.032481	0.028695	POST < PRE
1H-Purine-2,6-dione, 3,7-dihydro-3-methyl-	-3.136553	0.012139	POST < PRE
Indoleacetic acid	-3.283499	0.002439	POST < PRE
Pyruvic acid oxime	-3.329487	0.001231	POST < PRE
2-Hydroxyisocaproic acid	-3.506878	0.007983	POST < PRE
Octadecanoic acid, propyl ester	-4.297371	0.00107	POST < PRE
3-tert-Butylphenol	-4.432553	7.72E-06	POST < PRE
1-Docosene	-5.019577	2.74E-05	POST < PRE
L-Threonine	-5.809352	2.59E-06	POST < PRE

Table S10. OLP patients' fecal metabolites. Differentially abundant fecal metabolites between baseline (PRE) and after (POST) treatment quantifications ($p<0.05$). Numeric values represent fold changes (PRE/POST ratio) in logarithmic scale, p-values (adjusted for multiple testing), and interpretation of the difference (as direction), derived from linear mixed-effects regression models controlling for baseline covariates (age, sex, BMI, Vitamin D concentration, and disease severity).

Metabolite	log(POST/PRE)	P-value	Direction
Methyl alpha-D-glucofuranoside	2.67740103	0.00100791	POST > PRE
2-Isopropyl-5-methyl-1-heptanol	2.38481504	0.0432252	POST > PRE
Catechol	1.91974195	0.00836937	POST > PRE
1-Monooleoylglycerol	1.75458977	0.0432252	POST > PRE
2-Hydroxybutyric acid	1.339908	0.0081209	POST > PRE
Ser-Leu	1.32361766	0.0081209	POST > PRE
Behenic acid	1.15441589	0.00198752	POST > PRE
Xylonic acid	1.12756374	0.01286673	POST > PRE
13-Docosenamide	0.97124943	0.0081209	POST > PRE
Erythritol	0.96086879	0.0081209	POST > PRE
Adenine	0.85253514	0.0153543	POST > PRE
Niacinamide	0.8252831	0.03830803	POST > PRE
1-Octadecanol	0.5844023	0.0064093	POST > PRE
3-Octadecanone	0.5002835	0.01253438	POST > PRE
L-Glutamic acid	0.45789937	0.00836937	POST > PRE
Arachidic acid	0.31292843	0.01651599	POST > PRE
2,2,4-Trimethyl-1,3-pentanediol diisobutyrate	0.24068972	0.0153543	POST > PRE
3,5-di-tert-Butyl-4-hydroxybenzaldehyde	0.2373089	0.02162203	POST > PRE
Lactic Acid	-0.29565893	0.03128907	POST < PRE
Glyoxime	-0.32122361	0.03830803	POST < PRE
L-Threonine	-0.54107086	0.03128907	POST < PRE
p-Toluic acid	-0.83147077	0.00029991	POST < PRE
2-deoxy-erythro-pentonic acid	-0.85582426	0.0432252	POST < PRE
Cyclobutylamine	-0.8977678	0.03830803	POST < PRE
L-Lysine	-0.899596	0.00836937	POST < PRE
Acetin	-0.94541609	0.04147637	POST < PRE
Picolinic acid	-1.02247424	0.03128907	POST < PRE
2,6-Di-tert-butyl-4-hydroxy-4-methylcyclohexa-2,5-dien-1-one	-1.35672349	0.0000286	POST < PRE
N-Acetyltyrosine	-1.35962492	0.00836937	POST < PRE
1,5-Anhydroglucitol	-1.48951543	0.00836937	POST < PRE
3-Hydroxybutyric acid	-1.96512983	0.00094361	POST < PRE

Metabolite	log(POST/PRE)	P-value	Direction
D-(-)-Fructofuranose	-2.20143282	0.0081209	POST < PRE
Glycine	-2.27805273	0.02247818	POST < PRE
Pimelic acid	-2.49716876	0.0432252	POST < PRE

Oral and gut microbiome

Table S11: Summary quality statistics grouped by tissue of origin after removing features classified as Eukaryota and those lacking a taxonomic assignment at the phylum level.

Specimen	Total reads (Million)	Average reads per sample (SD) (Million)	Number of taxa	Unclassified genera
Saliva (S)	4.0	0.08 (0.07)	1338	133
Lesion (L)	6.8	0.14 (0.07)	1094	125
Non Lesion (NL)	4.5	0.09 (0.06)	1332	139
Feces (F)	4.6	0.09 (0.09)	1479	116

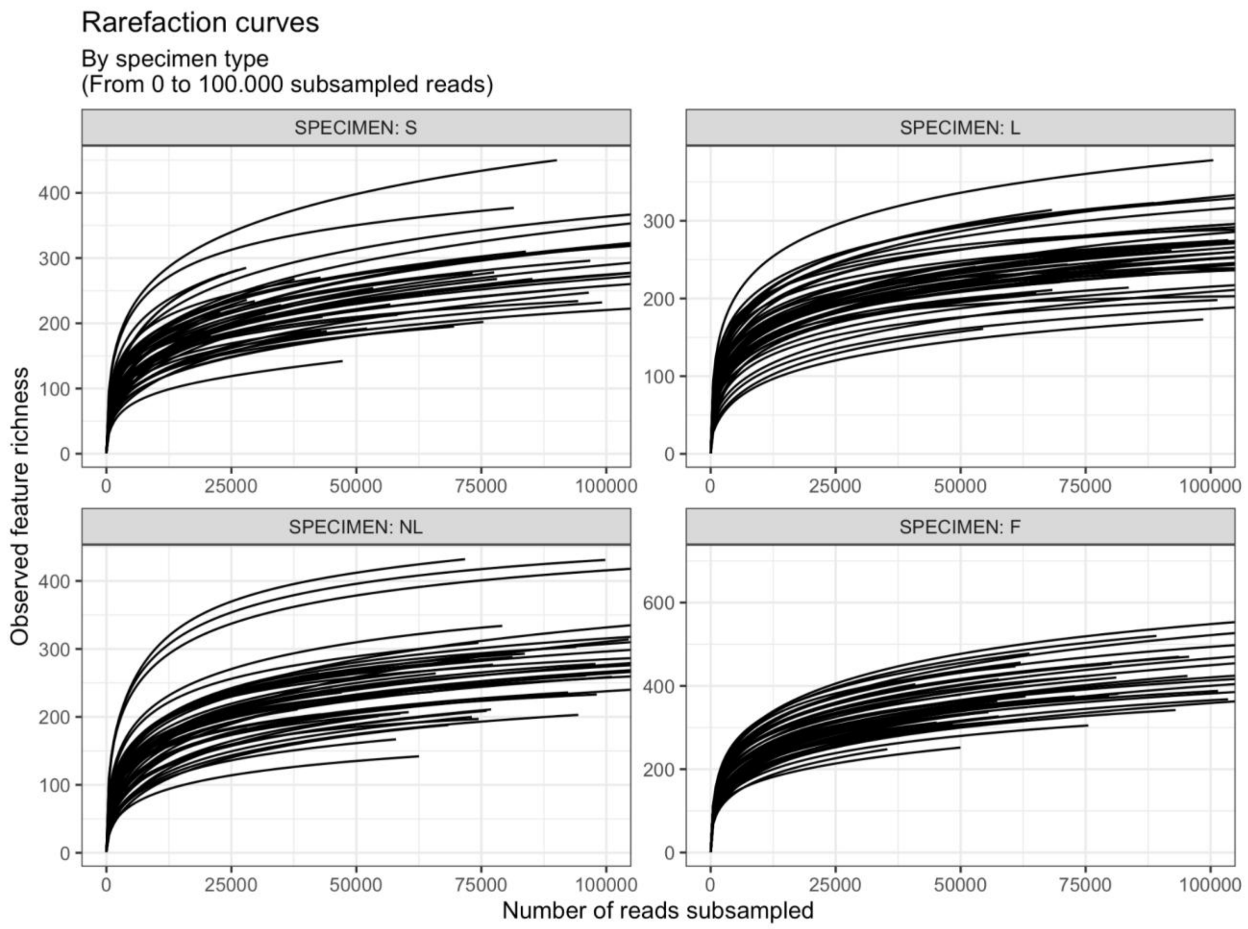

Figure S1: Rarefaction curves for each sample stratified by saliva (S), OLP-lesioned mucosa (L), non-lesioned healthy mucosa (NL), and feces samples (F). The x-axis is truncated at 100,000 subsampled reads for clarity.

Table S12: Total number and average number of features per sample (SD) before and after genus level agglomeration and prevalence filter.

	Before agglomeration and filter		After agglomeration and filter		
**Specimen**	**Total taxa**	**Average per sample (SD)**	**Total taxa**	**Average per sample (SD)**	**Retained Reads %**
Saliva (S)	1338	262.58 (62.66)	246	124.14 (21.79)	99.94
Lesion (L)	1094	264.54 (48.13)	214	121.1 (16.64)	99.95
Non Lesion (NL)	1332	263.1 (64.46)	235	121.92 (21.58)	99.86
Feces (F)	1479	399.3 (81.77)	291	163.5 (26.75)	99.74

Table S13: Relative abundances (%) for each phylum across saliva (S), OLP-lesioned mucosa (L), non-lesioned healthy mucosa (NL), and feces (F).

Phylum	S	L	NL	F
Bacillota	82.83	83.25	87.47	56.96
Actinomycetota	12.18	11.39	7.21	4.71
Candidatus Saccharibacteria	2.90	2.17	1.58	0.01
Pseudomonadota	0.80	1.01	1.62	3.65
Bacteroidota	0.77	1.34	1.25	32.79
Fusobacteriota	0.48	0.78	0.71	0.04
Campylobacterota	0.02	0.04	0.12	0.00
Chloroflexota	0.01	0.01	0.00	-
Euryarchaeota	0.01	0.01	0.00	0.24
Spirochaetota	0.01	0.00	0.01	-
Cyanobacteriota	0.00	0.00	0.00	-
Deinococcota	0.00	0.00	0.00	-
Synergistota	0.00	0.00	0.01	0.11
Verrucomicrobiota	0.00	-	-	1.46
Mycoplasmatota	-	0.00	0.00	0.00
Lentisphaerota	-	-	-	0.02

Table S14: Number of genera for each phylum across saliva (S), OLP-lesioned mucosa (L), non-lesioned healthy mucosa (NL), and feces (F).

	**S**	**L**	**NL**	**F**
Bacillota	101	78	83	170
Pseudomonadota	62	60	74	34
Actinomycetota	31	28	28	36
Bacteroidota	34	27	28	32
Fusobacteriota	6	6	7	4
Campylobacterota	3	4	4	1
Spirochaetota	2	3	3	0
Euryarchaeota	1	1	1	4
Synergistota	1	1	2	3
Verrucomicrobiota	1	0	0	4
Candidatus Saccharibacteria	1	1	1	1
Cyanobacteriota	1	2	1	0
Chloroflexota	1	1	1	0
Deinococcota	1	1	1	0
Mycoplasmatota	0	1	1	1
Lentisphaerota	0	0	0	1

Table S15: Adjusted alpha diversity metrics (number of genera, relative dominance of the most abundant genus, and Shannon diversity index) before (PRE) and after (POST) the 16-week intervention, stratified by specimen type: saliva (S), OLP-lesioned mucosa (L), non-lesioned healthy mucosa (NL), and feces (F). Values represent estimated marginal means (± standard error) obtained from linear mixed-effects models controlling for baseline covariates (age, sex, BMI, baseline Vitamin D concentration, and disease severity). Pairwise contrasts are reported for time (POST vs PRE) and across specimens (S vs L, S vs NL, L vs NL, S vs F, L vs F, NL vs F), with corresponding 95% confidence intervals. Statistically significant contrasts (Šidák and Benjamini–Hochberg adjusted p-value < 0.05) are shown in bold and marked with an asterisk.

		TIME		Contrast
**α-diversity**	**SPECIMEN**	**PRE**	**POST**	**POST - PRE**
**Number of genera**	S	118.64 ± 4.65	130.20 ± 4.65	11.56 (-5.70, 28.82,)
	L	118.80 ± 4.65	123.96 ± 4.65	5.16 (-12.10, 22.42)
	NL	121.40 ± 4.65	123.00 ± 4.65	1.60 (-15.66, -18.86)
	F	161.76 ± 4.65	165.80 ± 4.65	4.04 (-13.22, -21.30)
Between SPECIMEN contrasts	S - L	-0.16 (-17.42, 17.10)	6.24 (-11.02, 23.50)	
	S - NL	-2.76 (-20.02, 14.50)	7.20 (-10.06, 24.46)	
	L - NL	-2.60 (-19.86, 14.66)	0.96 (-16.30, 18.22)	
	**S - F**	**-43.12 (-60.38, -25.86)***	**-35.60 (-52.86, -18.34)***	
	**L - F**	**-42.96 (-60.22, -25.70)***	**-41.84 (-59.10, -24.58)***	
	**NL - F**	**-40.36 (-57.62, -23.10)***	**-42.80 (-60.06, -25.54)***	
**Relative dominance**	S	0.40 ± 0.02	0.40 ± 0.02	0.00 (-0.08, 0.08)
	L	0.38 ± 0.02	0.37 ± 0.02	-0.01 (-0.09, 0.06)
	NL	0.44 ± 0.02	0.44 ± 0.02	0.00 (-0.07, 0.08)
	F	0.16 ± 0.02	0.16 ± 0.02	-0.00 (-0.08, 0.07)
Between SPECIMEN contrasts	S - L	0.02 (-0.06, 0.09)	0.03 (-0.05, 0.11)	
	S - NL	-0.05 (-0.12, 0.03)	-0.05 (-0.12, 0.03)	
	**L - NL**	-0.06 (-0.14, 0.02)	**-0.08 (-0.15, -0.00)***	
	**S - F**	**0.24 (0.16, 0.31)***	**0.24 (0.17, 0.32)***	
	**L - F**	**0.22 (0.15, 0.30)***	**0.21 (0.13, 0.29)***	
	**NL - F**	**0.28 (0.21, 0.36)***	**0.29 (0.21, 0.36)***	
**Shannon's diversity**	S	2.18 ± 0.09	2.24 ± 0.09	0.06 (-0.21, 0.34)
	L	2.25 ± 0.09	2.28 ± 0.09	0.03 (-0.25, 0.31)
	NL	2.04 ± 0.09	2.02 ± 0.09	-0.02 (-0.30, 0.25)
	F	3.28 ± 0.09	3.31 ± 0.09	0.03 (-0.25, 0.31)
Between SPECIMEN contrasts	S - L	-0.07 (-0.35, 0.21)	-0.04 (-0.32, 0.24)	
	S - NL	0.14 (-0.14, 0.42)	0.23 (-0.05, 0.50)	
	L - NL	0.21 (-0.07, 0.49)	0.26 (-0.01, 0.54)	
	**S - F**	**-1.10 (-1.38, -0.82)***	**-1.07 (-1.35, -0.79)***	
	**L - F**	**-1.03 (-1.31, -0.75)***	**-1.03 (-1.31, -0.75)***	
	**NL - F**	**-1.24 (-1.52, -0.96)***	**-1.29 (-1.57, -1.02)***	
